# Genome-wide CRISPR screen reveals the synthetic lethality between BCL2L1 inhibition and radiotherapy

**DOI:** 10.26508/lsa.202302353

**Published:** 2024-02-05

**Authors:** Ling Yin, Xiaoding Hu, Guangsheng Pei, Mengfan Tang, You Zhou, Huimin Zhang, Min Huang, Siting Li, Jie Zhang, Citu Citu, Zhongming Zhao, Bisrat G Debeb, Xu Feng, Junjie Chen

**Affiliations:** 1 Department of Experimental Radiation Oncology, The University of Texas MD Anderson Cancer Center , Houston, TX, USA; 2 Department of Breast Medical Oncology, The University of Texas MD Anderson Cancer Center , Houston, TX, USA; 3 Morgan Welch Inflammatory Breast Cancer Clinic and Research Program, The University of Texas MD Anderson Cancer Center , Houston, TX, USA; 4 Human Genetics Center, School of Public Health, The University of Texas Health Science Center at Houston, Houston, TX, USA; 5 Center for Precision Health, School of Biomedical Informatics, The University of Texas Health Science Center at Houston, Houston, TX, USA; 6 Department of Pediatrics Research, Division of Pediatrics, The University of Texas MD Anderson Cancer Center , Houston, TX, USA; 7 Pancreas Center, First Affiliated Hospital of Nanjing Medical University, Nanjing, China; 8 Pancreas Institute, Nanjing Medical University, Nanjing, China

## Abstract

Radiation therapy (RT) is one of the most commonly used anticancer therapies. However, the landscape of cellular response to irradiation, especially to a single high-dose irradiation, remains largely unknown. In this study, we performed a whole-genome CRISPR loss-of-function screen and revealed temporal inherent and acquired responses to RT. Specifically, we found that loss of the IL1R1 pathway led to cellular resistance to RT. This is in part because of the involvement of radiation-induced IL1R1-dependent transcriptional regulation, which relies on the NF-κB pathway. Moreover, the mitochondrial anti-apoptotic pathway, particularly the BCL2L1 gene, is crucially important for cell survival after radiation. BCL2L1 inhibition combined with RT dramatically impeded tumor growth in several breast cancer cell lines and syngeneic models. Taken together, our results suggest that the combination of an apoptosis inhibitor such as a BCL2L1 inhibitor with RT may represent a promising anticancer strategy for solid cancers including breast cancer.

## Abstract (precis)

Unbiased whole-genome CRISPR/Cas9 screening reveals that loss of BCL2L1 shows synthetic lethality with radiation, which is further confirmed with the use of BCL2L1 inhibitor. Thus, radiation plus inhibitors targeting BCL2 family proteins is a promising combination therapy for cancers.

## Introduction

Radiation therapy (RT) is a widely used and essential treatment modality in cancer (Barton et al, 2014); ∼50% of all cancer patients receive RT either as a standalone treatment or in combination with surgery or chemotherapy (Delaney et al, 2005; Kocakavuk et al, 2021). Although radiotherapy has been proven to be effective in numerous cases, substantial challenges persist, which center on avoiding radiation resistance and enhancing radiation sensitivity (Kim et al, 2015). Significant endeavors have been devoted to unravel the mechanisms involved in the acquisition of radioresistance (Shimura et al, 2010; Barker et al, 2015). Indeed, prior studies revealed multiple factors dedicated to these phenomena, encompassing heightened DNA damage responses, the quiescent nature of cancer stem cells, dysregulated signaling pathways such as PI3K/mTOR and NF-ƙB, epithelial-to-mesenchymal transition (EMT), and the impact of tumor microenvironment including hypoxia (Fokas et al, 2012; Gray et al, 2019; Sato et al, 2019). To achieve better tumor control, it is crucial to identify biomarkers that can help overcome radiation resistance and/or improve radiation sensitization (Begg et al, 2011). The use of combination therapies, such as RT combined with chemotherapy or targeted agents, could potentially enhance the efficacy of radiation therapy.

With the advancement of genome-wide clustered regularly interspaced short palindromic repeats (CRISPR)/Cas9 screening and analytical tools, pooled genome-wide CRISPR/Cas9 screening has emerged as a potent and highly sensitive approach for elucidating genetic interactions in human cells (Hart et al, 2015). CRISPR/Cas9 has been extensively employed to unveil synthetic lethal relationships with anticancer drugs, aiming to identify genetic vulnerabilities and explore new combination therapies for potential clinical trials (Shalem et al, 2014; Huang et al, 2023). In this study, we conducted single guide RNA (sgRNA) screening in conjunction with high-dose ionizing radiation treatment in MCF10A cells, with the goals of uncovering genes and pathways associated with radiation sensitivity and resistance.

BCL2-like protein 1 (BCL2L1) belongs to the anti-apoptotic BCL2 protein family (Morales-Martínez & Vega, 2022). BCL2 family proteins are key regulators responsible for maintaining the balance between cell life and death. They control mitochondria membrane permeabilization, allowing the release of apoptogenic factors, including cytochrome C (Cory & Adams, 2002). Structurally, all BCL2 family proteins contain, at least one of, four conserved regions of a homologous sequence called the BCL2 homology (BH) domains 1–4 and a hydrophobic C-terminal region acting as a transmembrane anchor. Among these regions, the BH1 and BH2 domains are essential for dimerization with pro-apoptotic proteins, whereas the BH3 domain appears in all BCL2 family members and is critically important for the interaction between pro-apoptotic and anti-apoptotic proteins (Pistritto et al, 2016). Accordingly, the BCL2 clan is divided into pro-survival members, such as BCL2, BCL2L1, BCL-W, MCL-1, and A1, and pro-apoptotic members, such as BAX and BH3-only proteins, which are required to initiate apoptosis. Compared with the other family members, both *BCL2L1* and *BCL2* have a longer sequence connecting the N-terminal region containing the BH4 domain and the BH3 domain (Lee & Fairlie, 2019). BCL2 family genes are highly related to tumor development and prognosis among various cancers, and *BCL2L1* is one of the most common amplified genes in cancer. Abnormal genetic expression of the BCL2 family members has been associated with worsened outcomes in breast cancer (Merino et al, 2017; Wang et al, 2020a) and lung cancer (Qin et al, 2019). Previous studies indicated BCL2 to be related to paclitaxel resistance in ovarian cancer (Yin et al, 2021), and up-regulation of *BCL2L1* has been implicated in platinum and PARP inhibitor resistance in ovarian cancer (Guo et al, 2021). Thus, *BCL2L1* may contribute not only to tumor development but also to therapy resistance.

In this study, we found that loss of *BCL2L1* showed synthetic lethal interaction with irradiation via unbiased whole-genome CRISPR/Cas9 screening, which was further confirmed with BCL2L1 inhibitors in vitro and in vivo. Thus, our results provide a promising and rational combination, that is, radiation plus BCL2 inhibitors, for cancer treatment.

## Results

### Whole-genome CRISPR screens in breast epithelial cells with a single high dose of radiation

To identify genetic vulnerabilities to irradiation in breast cancer, we conducted unbiased whole-genome CRISPR/Cas9 screens in MCF10A breast epithelial cells. The reason we chose a mammary epithelial cell line MCF10A rather than a human breast cancer cell line is that normal cells may not exhibit as much heterogeneity and genetic alternations as tumor cell lines, which allows us to explore all possible genetic determinants of cellular response to radiation therapy. In addition, regardless the cell line we chose for this screen, subsequent validation experiments using additional cell lines, especially cancer cells, are needed to confirm our conclusions.

**Figure 1.**
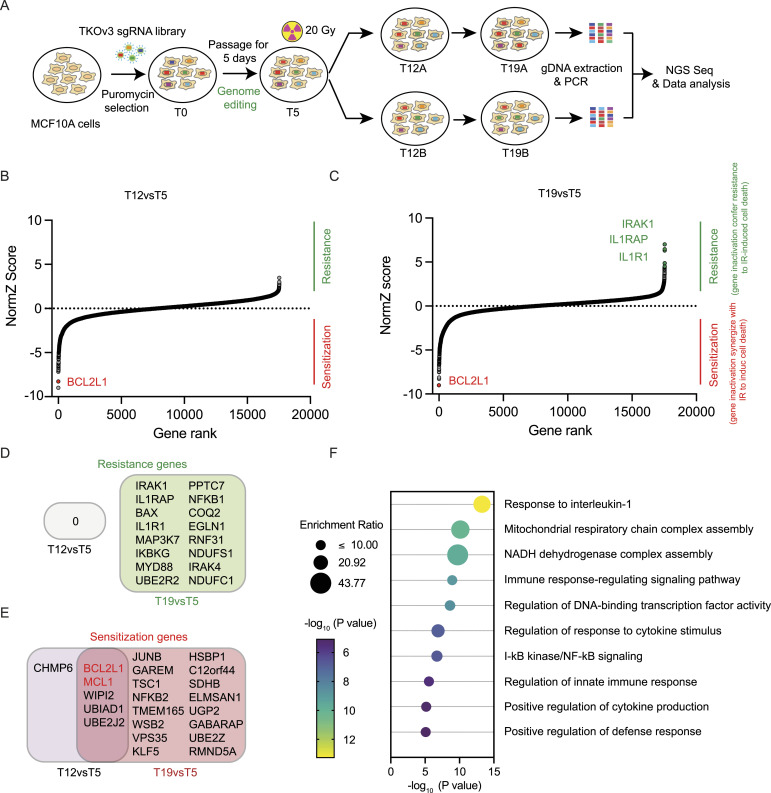
Genome-wide CRISPR/Cas9 screens in MCF10A cells with a single high dose of radiation. **(A)** Schematic of the workflow for CRISPR screen in MCF10A cells treated with radiation and the TKOv3 whole-genome gRNA library. Cells infected with the TKOv3 library virus were cultured and passaged after puromycin selection. At day 5, all cells were treated with 20 Gy of radiation and then divided into two biological replicates. We collected cells at day 5 (T5), day 12 (T12), and day 19 (T19). Genomic DNA extracted from different groups of cells was amplified, sequenced, and analyzed. **(B, C)** Ranking of the coessential genes based on DrugZ analysis of the screen results. **(B)** sgRNA read counts from 7 d after radiation (cells at T12) were compared with the read counts from cells at T5 without radiation. **(C)** sgRNA read counts from 14 d after radiation (cells at T19) were also compared with the read counts from cells at T5 without radiation. Positive NormZ scores suggest that these sgRNAs were enriched in the postradiation group, indicating that they target radiation-sensitizing genes. Likewise, negative NormZ scores suggest that these sgRNAs were depleted in the postradiation groups, indicating that they target radiation-resistant genes. **(D)** Overlay of the genes whose depletion conferred resistance to radiation between the early (12 d after IR) and late (19 d after IR) periods. **(E)** Overlay of the genes whose loss led to radiation ensitization between the early (12 d after IR) and late (19 d after IR) periods. **(F)** Top 10 significantly enriched Gene Ontology (GO) Biological Process terms (*P* < 0.001) with the high-confidence candidate genes whose loss of function led to radiation resistance in MCF10A cells.

**Figure S1.**
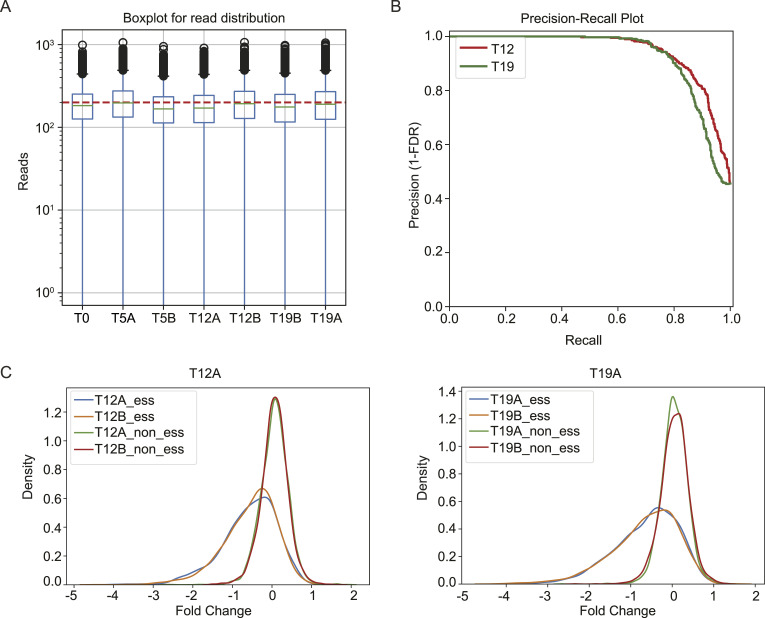
Quality control of CRISPR screen. **(A)** Deep-sequencing reads distribution for MCF10A cell with radiation treatment samples. The distributions of each sgRNA are presented. **(B)** Precision-recall plots of essential genes identified from these screens. The dashed line marks the 5% FDR. **(C)** Changes in essential and nonessential genes during the CRISPR screens. Comparing time point 12 (T12A and T12B, two technical replicates) with 5 (T5) and time point 19 (T19A and T19B, two technical replicates) with 5 (T5), sgRNAs targeting essential genes changed dramatically, but no change in sgRNAs targeting nonessential genes was observed.

The CRISPR screen workflow is shown in Fig 1A. Briefly, cells were infected with the Toronto KnockOut (TKOv3) sgRNA library virus as previously described (Wang et al, 2022). Then cells were cultured and passaged for about 20 doublings after puromycin selection. At day 5, all cells were treated with 20 Gy of irradiation. Genomic DNA extracted from different groups of cells was amplified, sequenced, and analyzed. We performed bioinformatic analysis to confirm that the screen data were reliable (Fig S1A–C). Genes were then ranked according to their NormZ scores after DrugZ analysis (Fig 1B and C, Table S1). Positive NormZ scores (sgRNA enrichment after radiation) suggested genes whose loss confers radiation resistance in MCF10A cells, and negative NormZ scores (sgRNA depletion after radiation) indicated genes whose loss leads to radiation sensitization.

Table S1 CRISPR screen results and ranking by DrugZ analysis.

To gain a deeper understanding of genes associated with radiation, we collected cells at two distinct time points: 1 wk after radiotherapy (RT) at day 12, defined as the early period, and 2 wk after RT at day 19, considered as the late period. We conducted a comparative analysis between day 12 (early after treatment) and day 19 (late) with the baseline data before radiation at day 5. In the early period, no genes displayed significantly positive NormZ scores. However, *BCL2L1* ranked prominently in the negative direction (Fig 1B). In the late period, top-ranking positive NormZ scores were seen for interleukin-1 receptor (*IL1R1*), interleukin-1 receptor accessory protein (*IL1RAP*), and interleukin-1 receptor–associated kinase 1 (*IRAK1*) (Fig 1C). Notably, *BCL2L1* exhibited negative NormZ scores in the late period, indicating that the inhibition of *BCL2L1* sensitizes cells to radiation.

Next, we used FDR values less than 0.1 as the criteria for inclusion in a list of candidate genes whose loss potentially conferred resistance or sensitization to RT. As shown in Fig 1D, no gene was associated with resistance early after radiation. Thus, we collected the candidate genes associated with late response (i.e., day 19 compared with day 5). Genes whose loss was associated with resistance were *IRAK1*, *IL1RAP*, *IL1R1*, *MAP3K7*, *IRAK4*, *NFKB1*, and others. By performing functional enrichment analysis using Gene Ontology, we found that these candidate genes are associated with response to interleukin-1, mitochondrial respiratory chain complex assembly, IκB kinase/NF-κB signaling, and others (Fig 1F; Table S2). We then overlaid genes (FDA < 0.1) associated with sensitization to radiation in the early and late periods and found that *BCL2L1* and *MCL1* were on the top of both lists (Fig 1E), which indicates that both genes displayed synthetic lethal interaction with RT. Taken together, these data suggest that loss of *IL1R1* or *IL1RAP* leads to cellular resistance to a single high dose of radiation, whereas inhibition of *BCL2L1* or *MCL1* causes increased sensitivity to radiation.

Table S2 GO pathway enrichment analysis of candidate genes revealed by RNA-seq data.

### Loss of IL1R1 or IL1RAP leads to radiation resistance

**Figure 2.**
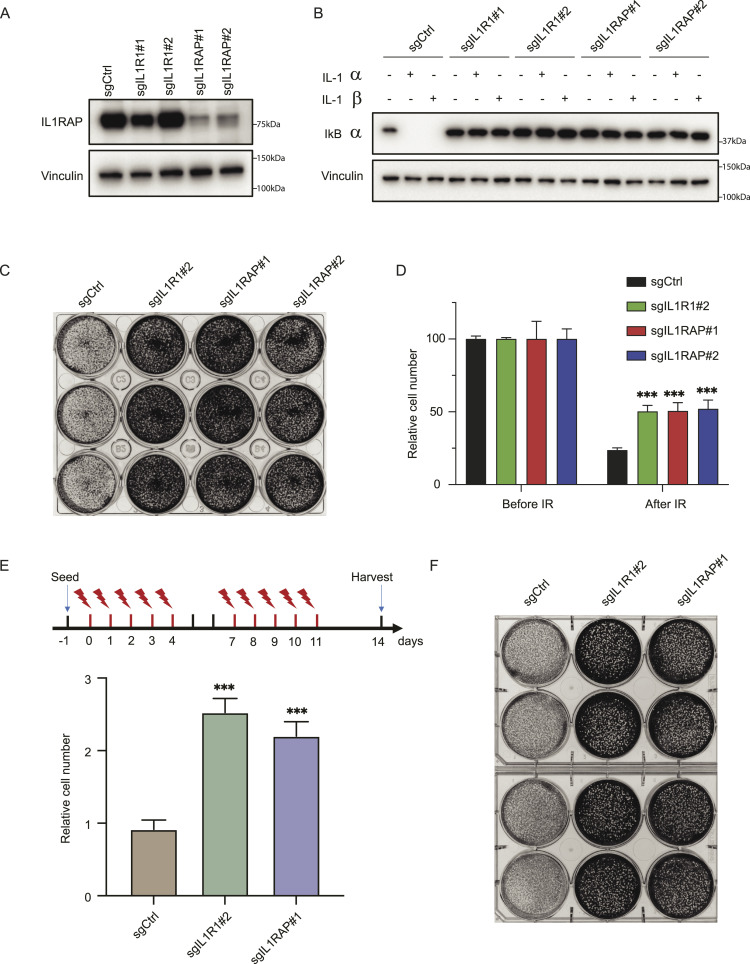
Loss of IL1R1 or IL1RAP leads to radiation resistance. **(A)** Western blots were performed to determine the efficacy of sgRNAs targeting IL1RAP. MCF10A cells were infected with lentiviruses expressing the indicated sgRNAs. After treatment with puromycin for 48 h, cells were collected and lysed by sodium dodecyl sulfate loading buffer. IL1RAP and vinculin were blotted with indicated antibodies. **(B)** Western blots to confirm the efficacy of sgRNAs targeting *IL1R1* and *IL1RAP*. MCF10A cells were infected with lentivirus expressing indicated sgRNAs and selected with puromycin. Cells were treated with IL-1α (10 ng/ml) or IL-1β (10 ng/ml) for 0.5 h and then collected. Cell lysates were prepared and blotted with indicated antibodies. **(C)** Cell growth assays were performed using MCF10A cells infected with lentivirus expressing sgRNAs targeting *IL1R1* or *IL1RAP* and under a single high dose of radiation treatment. 1 × 10^5^ cells were seeded in a 12-well plate and incubated 24 h. Cells were irradiated with a single high dose of 20 Gy and maintained for 14 d. Cells were then collected and counted with an automated cell counter (TC20, Bio-Rad). Experiments were performed in triplicate with three biological replicates. Representative images and results are shown. **(C, D)** Quantification of relative survival in cell growth assay presented in (C). *t* tests were performed to estimate differences between two groups, and the data are presented as means ± SD. **P < 0.01, ***P < 0.001. **(E)** Results of crystal violet staining and quantification assay. MCF10A cells infected with indicated sgRNAs were treated with a conventional radiation schedule, which is 2 Gy per day for 5 d, 2 d off, and then continued for another 5 d (scheme on top). 2 × 10^5^ cells were seeded in six-well plates and 24 h later treated with indicated conditions. After 14 d, cells were stained with crystal violet solution, and relative survival was quantified by the Synergy multimode microplate reader (histogram on the bottom). The data are presented as means ± SD. **P < 0.01; ***P < 0.001 (*t* test). **(F)** Cell growth assays were performed using MCF10A cells infected with lentivirus expressing indicated sgRNAs and under conventional radiation treatment. **(E)** 2 × 10^5^ cells were seeded in a six-well plate, and 24 h later, cells were irradiated with the same conditions as in (E). Cells were fixed and stained. Experiments were performed in triplicate with three or four biological replicates. Representative images and results are shown.

To test the results obtained from the screen, we used sgRNAs to knock out *IL1R1* and *IL1RAP* in MCF10A cells. As shown in Fig 2A, Western blot analysis confirmed the depletion of IL1RAP in sgIL1RAP#1 and sgIL1RAP#2 cells. Unfortunately, despite testing several antibodies, we were unable to find a suitable antibody for IL1R1. To circumvent the issue of lacking specific antibodies recognizing IL1R1, we incubated wild-type cells and sgRNA-mediated KO cell lines (sgIL1-R1#1, sgIL1-R1#2, sgIL1RAP#1, and sgIL1RAP#2) with IL-1α or IL-1β. As depicted in Fig 2B, wild-type cells showed IκBα degradation, which was induced by IL-1α or IL-1β, and all KO cells exhibited resistance to IκBα degradation after treatment with IL-1α or IL-1β, confirming the successful establishment of *IL1R1* and *IL1RAP* KO in MCF10A cells.

To validate the hypothesis that KO of IL1R1 or IL1RAP induces radiation resistance, we delivered a single high dose of RT and conventional RT to the control and KO cell lines. Cell proliferation assays indicated that *IL1R1* and *IL1RAP* KO cells exhibited significantly higher radiation resistance compared with wild-type cells (Fig 2C and D). Consistently, after conventional RT, the KO cells demonstrated significantly improved survival compared with wild-type cells (Fig 2E and F). In summary, our data suggest that *IL1R1* and *IL1RAP* play a critical role in RT resistance.

### NF-κB acts downstream of IL1R1 and IL1RAP in radiation resistance

**Figure 3.**
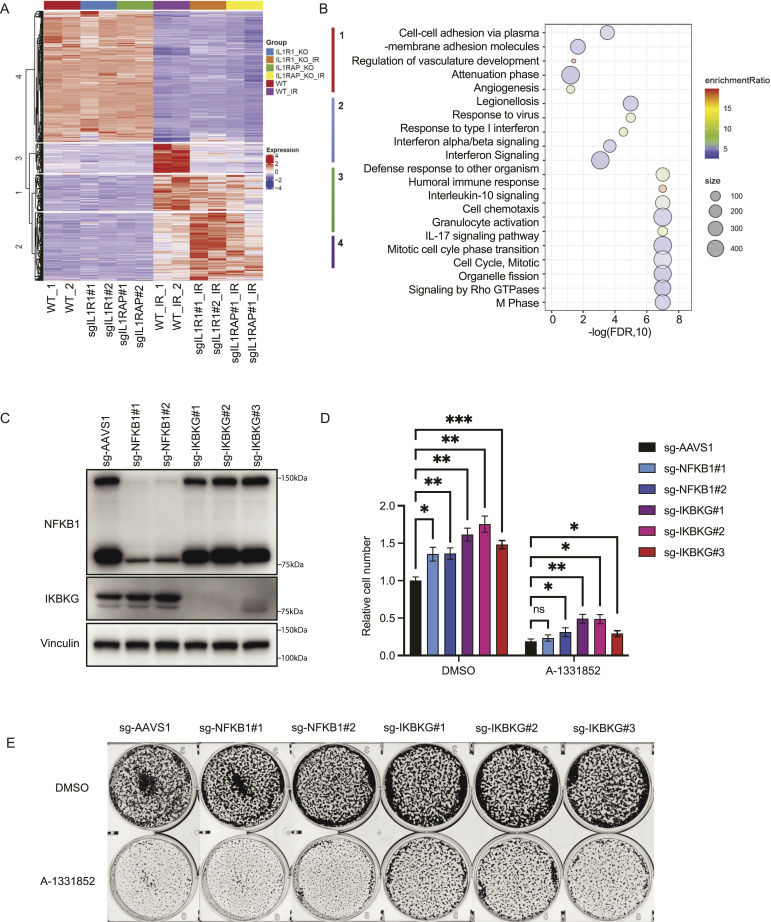
NF-κB is the downstream effector by which IL1R1 and IL1RAP induce radiation resistance. **(A)** Hierarchical clustering analysis in MCF10A cells of different groups with indicated treatments. MCF10A cells were infected with sgRNAs targeting *IL1R1* or *IL1RAP*. RT groups were treated with a single high dose of 20 Gy of irradiation. Each group has two biological repeats. **(B)** Kyoto Encyclopedia of Genes and Genomes pathway enrichment analysis of differentially expressed genes (z-score scaled) from irradiation groups. **(C)** MCF10A cells were infected with lentiviruses containing LentiCRISPRv2 sgControl or two independent sgRNAs targeting NF-κB and three independent sgRNAs targeting *IKBKG*. 24 h later, cells were selected with puromycin (2 mg/ml) for 3 d and Western blotted with the indicated antibodies. **(C, D)** Same conditions as those described in **(C)**, except that cells after puromycin selection were seeded at 2 × 10^5^ per well in a six-well plate with four replicates. 24 h later, cells were treated with a single high dose of 20 Gy radiation and BCL2L1 inhibitor A-1331852 (1 μM) for 10 d. Luminescence signals were detected according to the standard protocol. The data are presented as means ± SD. **P < 0.01; ***P < 0.001 (*t* test). **(D, E)** Same condition as that described in **(D)**, except cells were seeded at 2 × 10^5^ per well in a six-well plate with three replicates. 24 h later, cells were treated with a single high dose of 20 Gy radiation and BCL2L1 inhibitor A-1331852 (1 μM) for 10 days. Cells were fixed, stained, and further analyzed by ImageJ. Representative images are shown.

To investigate the potential mechanisms underlying the roles of *IL1R1* and *IL1RAP* in radiation resistance, we conducted mRNA sequencing analysis using wild-type MCF10A cells and *IL1R1*-KO and *IL1RAP*-KO cells. The parental cells and KO cells were subjected to RT or left untreated. At an FDR <0.05 and fold change >2, we identified differentially expressed genes in each group (Table S3). Clustering analysis indicate that cluster 1, cluster 2, and cluster 4 are genes changed in all cells regardless of their genotypes (i.e., WT, IL1R1-KO, and IL1RAP-KO) after RT treatment when compared with control cells without RT treatment (Fig 3A). Genes from cluster 1 and cluster 2 were up-regulated after RT, whereas genes in cluster 4 were down-regulated in cells treated with RT.

Table S3 DEGs of each comparison obtained from RNA-seq data.

As we were interested in exploring the potential mechanisms underlying the roles of *IL1R1* and *IL1RAP* in radiation resistance, we focused on cluster 3, which contains genes that were significantly down-regulated in IL1R1-KO and IL1RAP-KO cells when compared with those in wild-type cells. Subsequently, we performed pathway enrichment analysis of these differentially expressed genes using the Kyoto Encyclopedia of Genes and Genomes pathway annotations, as illustrated in Fig 3B and Table S4. The analysis highlighted five pathways predominantly enriched in cluster 3, which may contribute to radiation resistance in these KO cells. These pathways include the human immune response pathway, the IL-10 signaling pathway, the cell chemotaxis pathway, the granulocyte activation pathway, and the IL-17 signaling pathway. These enriched pathways are all related to the NF-κB kinase pathway (Baeuerle & Henkel, 1994; Dunn et al, 1994; Ferrè et al, 2010; Tamassia et al, 2010; Serasanambati & Chilakapati, 2016; Swaidani et al, 2019), which suggests that the down-regualtion of NF-κB may be at least one of the mechanisms by which loss of IL1R1 or IL1RAP1 leads to radiation resistance. These data also indicate that radiation treatment led to transcriptional up-regulation of a set of genes in an IL1R1- and IL1RAP-dependent manner, which may account for, at least in part, IL1R1/IL1RAP–mediated radiation sensitivity.

Table S4 KEGG pathway enrichment analysis of each comparison.

To test this hypothesis, we knocked out IKBKG and NFKB1 in MCF10A cells (Fig 3C). Indeed, both NFKB1-KO and IKBKG-KO cells exhibited significant radiation resistance when compared with wild-type cells (Fig 3D and E). Moreover, we identified many components of this pathway in our screen (Fig 1D), including not only IL1R1/IL1RAP and IKBKG/NF-kB but also MYD88 and IRAK1/4, which are known to be involved in the signaling pathway from IL1R1/IL1RAP to NF-kB. These data together support that knock out of this pathway leads to radiation resistance in MCF10A cells.

### BCL2L1 inhibitor sensitizes cells to radiation

The objective of our screening is to pinpoint vulnerabilities related to radiation. Based on the screen results, we identified that loss of IL1R1/IL1RAP induces radiation resistance through the inactivation of the NF-κB signaling pathway. However, it is challenging to overcome the considerable toxicity to normal tissue by activation of IL1R1/IL1RAP through IL-1 (Lierova et al, 2018). Thus, we shifted our attention to genes whose loss induces radiation sensitivity because inhibition of a gene and/or a pathway offers a more feasible approach to enhance radiation sensitivity.

**Figure 4.**
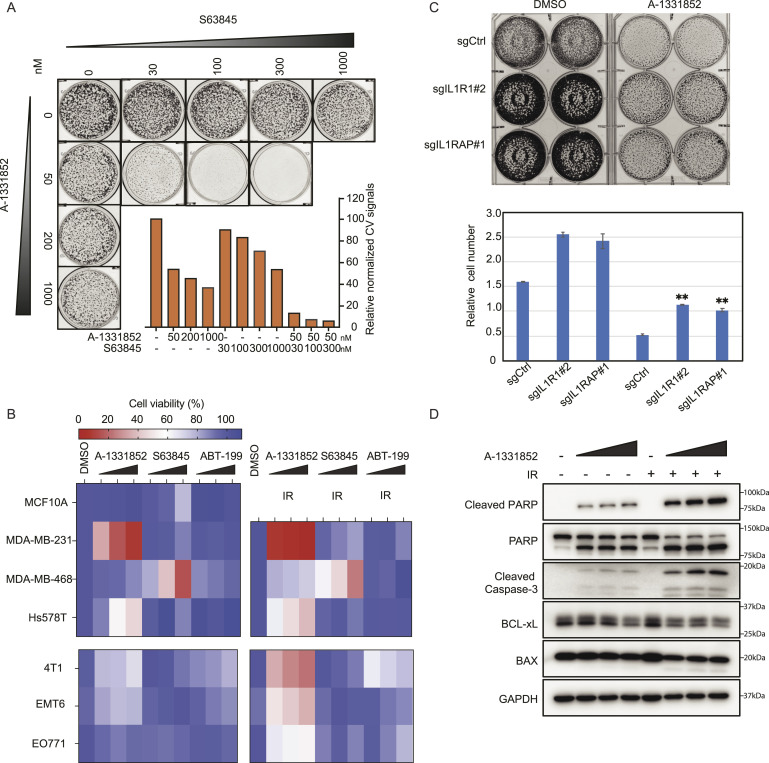
BCL2L1 inhibitor sensitizes cells to radiotherapy. **(A)** 1 × 10^5^ MCF10A cells were seeded in 12-well plates. 24 h later, cells were treated with a single high dose of 20 Gy irradiation and inhibitor A-1331852 or S63845 with indicated concentrations for 10 d. Cells were stained with crystal violet solution, and relative survival was quantified by the Synergy multimode microplate reader. Experiments were performed in triplicate with three biological replicates. Shown are representative images and results (top) and quantification of crystal violet staining assay (bottom). *t* tests were performed to estimate differences between two groups, and the data are presented as means ± SD. **P < 0.01, ***P < 0.001. **(B)** (Top) MCF10A, MDA-MD-231, MDA-MB-468, and Hs578T cells were treated with the three inhibitors under normal culture conditions or with irradiation. A-1331852 concentrations were 0, 50, 200, and 1,000 nM; those of S63845 were 100, 300, and 1,000 nM; those of ABT-199 were 100, 250, and 1,000 nM. 1 × 10^3^ cells were seeded per well in a 96-well plate with four replicates; 24 h later, cells were treated with radiation at 10 Gy for MDA-MD-231 and 20 Gy for MDA-MB-468 and Hs578T. (Bottom) 4T1, EMT6, and EO771 cells were treated with the three inhibitors under normal culture conditions or with irradiation. After cells were maintained for 5 d, luminescence signals were detected according to the standard protocol. The data are presented in heatmaps. **(C)** MCF10A cells were transfected with sgRNAs constructs targeting *IL1R1* or *IL1RAP*. 72 h after puromycin selection, 2 × 10^5^ cells were seeded in six-well plates, and after 24 h, cells were treated with a single high dose of 20 Gy radiation and A-1331852 (1 μM) for 10 d. Cells were stained with crystal violet solution, and relative survival was quantified by the Synergy multimode microplate reader. Shown are representative images and results (top) and quantification of crystal violet staining assay (bottom). The data are presented as means ± SD, and *t* tests were performed to estimate differences between two groups. **P < 0.01, ***P < 0.001. **(D)** MCF10A cells were treated with A-1331852 without or with radiation. Cells were collected and lysed for blotting with indicated antibodies.

Indeed, our results indicate that loss of BCL2L1 or MCL1 showed synthetic lethality with RT (Fig 1). BCL2L1 and MCL1 are well studied BCL2 family proteins with several candidate inhibitors. A-1331852 is a selective BH3 mimetic specifically targeting BCL2L1 (also called BCL-xL) protein (Wang et al, 2020b); this agent has the potential to enhance the efficacy of docetaxel in solid tumors (Leverson et al, 2015). S63845 binds specifically and with high affinity to the BH3-binding groove of MCL1 and has been an effective treatment in preclinical models of TNBC (Merino et al, 2017). Hence, to further investigate the roles of BCL2 family proteins in radiation sensitization, we treated MCF10A cells with different concentrations of the BCL2L1 inhibitor A-1331852 and the MCL1 inhibitor S63845 in combination with 20 Gy of irradiation. As shown in Fig 4A, inhibiting BCL2L1 and MCL1 increased radiation sensitization, which is consistent with our screening results.

After conducting our initial screen in MCF10A cells and verifying the results as mentioned above, we aimed to determine the combined effects of these BCL2 family inhibitors and radiotherapy (RT) in cancer cell lines. For this purpose, we selected three human breast cancer cell lines—MDA-MB-231, MDA-MB-468, and Hs578T—along with three murine breast cancer cell lines, namely, 4T1, EMT6, and EO771, to validate the observed effects of these combination therapies. In addition to the BCL2L1 and MCL1 inhibitors, we also included ABT-199, an FDA-approved selective BCL2 inhibitor. ABT-199 has been shown to enhance the efficacy of docetaxel in solid tumors (Leverson et al, 2015). As depicted in Fig 4B, without RT, only the BCL2L1 inhibitor and the MCL1 inhibitor induced cell death in these cell lines, whereas treatment with ABT-199 did not lead to obvious cell death in any of the four cell lines. However, under RT, MDA-MB-231, MDA-MB-468, and Hs578T cells exhibited significantly increased cell death when exposed to these inhibitors targeting BCL2 family proteins. Similar phenotypes were also observed in murine breast cancer cell lines 4T1, EMT6, and EO771 (Fig 4B). Briefly, in the absence of RT, no significant cell death was observed upon treatment with the three inhibitors. However, these cell lines exhibited increased cell death in response to radiation in combination with these inhibitors. These findings further support the notion that inhibitors targeting BCL2 family proteins including BCL2L1 enhance the effectiveness of RT.

Based on our screening results, loss of *IL1R1* or *IL1RAP* induces radiation resistance. To investigate whether the inhibition of BCL2L1 could reverse the RT resistance observed in *IL1R1*-KO and *IL1RAP*-KO cells, we treated these KO cells with the BCL2L1 inhibitor A-1331852 under radiation conditions. Remarkably, A-1331852 significantly enhanced cell death in *IL1R1*-KO and *IL1RAP*-KO cells compared with RT alone (Fig 4C). Furthermore, we examined the effects of the BCL2L1 inhibitor A-1331852 on NFKB1-KO and IKBKG-KO cells as these knocked out proteins act downstream of IL1R1 and IL1RAP according to our previous findings. As anticipated, A-1331852 effectively reversed the radiation resistance observed in these KO cells (Fig 3D and E). These results indicate that the BCL2L1 inhibitor may also be effective when used to treat cells displaying radiation resistance.

BCL2L1 participates in regulating apoptosis and cell death (Hagenbuchner et al, 2010; Tait & Green, 2013). To further confirm the mechanism by which inhibiting BCL2L1 enhances radiation sensitivity, we examined the activation of the apoptosis pathway after radiation. Indeed, treatment with A-1331852 led to increases in cleaved-PARP1 and cleaved caspase-3 after radiation (Fig 4D), suggesting that the BCL2L1 inhibitor promotes radiation sensitivity by inducing apoptosis in these cells.

### Combination of BCL2L1 inhibitor with RT inhibits breast cancer tumor growth

**Figure 5.**
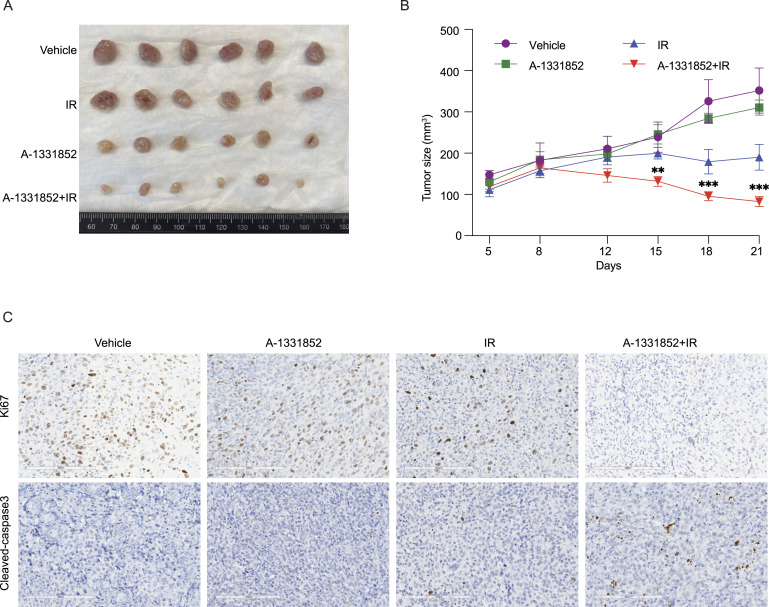
Combination of BCL2L1 inhibitor with radiation therapy decreases breast cancer tumor growth. **(A)** Briefly, 1 × 10^5^ 4T1 cells (mixed with Matrigel in 1:1 ratio) were subcutaneously injected into the right flank of 6-wk-old female BALB/cJ mice. Mice were randomly assigned to four groups (n = 8) and were treated with vehicle control (phosphate-buffered saline), radiation (a single dose of 5 Gy), inhibitor A-1331852, or the combination treatment. 25 kg/mg A-1331852 was given by oral gavage daily for 2 wk except for 2 d without treatment at the end of each week. Tumor sizes were measured twice a week starting at 6 d after injection. Representative tumors in each group were photographed at the endpoint. **(B)** The tumor volume (V) was calculated by the formula V = 1/2 × length × width^2^. Tumor growth curves of different treatment groups are presented. The tumor volumes shown at each point were the average from six tumors. ***P < 0.001 (unpaired *t* test). **(A, C)** Ki-67 (proliferation) and cleaved caspase-3 (apoptosis) staining were conducted in tumors shown in (A).

To validate our hypothesis in a preclinical setting, we conducted experiments using a 4T1 syngeneic model. This model, being immunocompetent, may be analogous to that of human patients because increasing evidence suggests that immune responses also contribute to the efficacy of radiation treatment in humans (Hekim et al, 2015; Walle et al, 2018). We assessed tumor growth after treatment with A-1331852, radiation, or the combination. Treatment with A-1331852 alone did not significantly suppress tumor growth compared with the control group. However, the radiation group showed smaller tumor sizes compared with the control group (Fig 5A). Importantly, the combination therapy of A-1331852 and radiation had a significantly greater effect in suppressing tumor growth compared with either treatment alone (Fig 5B). Furthermore, no obvious side effects were observed in the animals treated with the combination therapy.

Consistent with our in vitro observations, we detected a high level of apoptosis, as indicated by cleaved caspase-3 staining, in the tumors treated with the combination therapy. In contrast, the number of Ki-67–positive cells, a marker of proliferation, decreased in the combination group (Fig 5C). These findings further support our hypothesis that the BCL2L1 inhibitor sensitizes breast cancer cells to RT in an in vivo model.

Overall, the results obtained from our preclinical animal model provide strong evidence for the efficacy of this combination therapy in suppressing tumor growth, inducing apoptosis, and reducing proliferation. These findings highlight the potential of inhibitors targeting BCL2 family proteins as radiosensitizers for the treatment of breast and other cancers.

## Discussion

**Figure S2.**
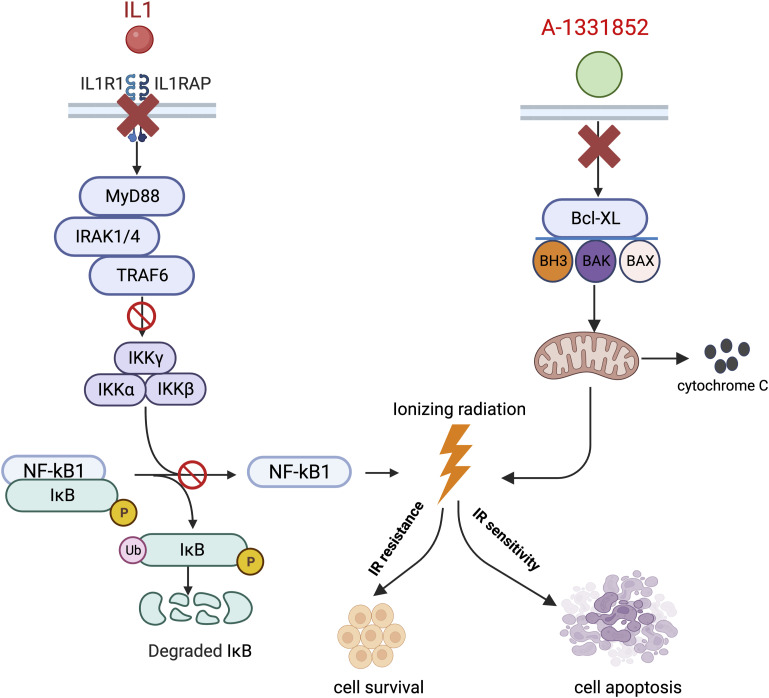
A proposed model. Knockout of IL1RA/IL1RAP inhibits the NF-kB pathway, leading the induction of cell radiation resistance. In addition, the BCL2L1 inhibitor A-1331852 induces radiation sensitivity through the activation cell apoptosis.

In this study, we performed genome-wide CRISPR/Cas9 screening to determine cellular response to RT. MCF10A cells were chosen for this screen because we speculated that the use of these non-tumorigenic cells may reveal the entire genetic determinants of cellular response to radiation. We showed that KO of IL1R1, IL1RAP, or their downstream effectors resulted in cellular resistance to RT. The mechanism underlying this radiation resistance is likely because of radiation-induced transcriptional changes, which rely in part on an IL1R1/IL1RAP-NF-κB–dependent signaling pathway, as revealed by RNA sequencing analysis. Moreover, we uncovered BCL2 family proteins, including BCL2L1 and MCL1 as potential mechanisms for radiation resistance not only in MCF10A cells but also in multiple human and murine breast cancer cell lines. Furthermore, treatment with inhibitors targeting these proteins resulted in radiation sensitization both in vitro and in vivo, likely by enhancing apoptosis after RT. Thus, our findings reveal that inhibitors targeting BCL2 family proteins can be used in combination with radiation to augment the efficacy of RT (Fig S2).

RT plays a crucial role in the comprehensive management of cancer and is an integral part of many treatment plans (Brunt et al, 2020). However, radiation resistance remains a significant challenge in clinical practice, highlighting the need to identify biomarkers that can help overcome this problem. To this end, genome-wide CRISPR/Cas9 screens have been extensively conducted to uncover genetic vulnerabilities that may be exploited to boost the efficacy of RT. For instance, Durocher and colleagues conducted 31 CRISPR-Cas9 screens against 27 genotoxic agents, including radiation in retinal pigment epithelium-1 cells (Olivieri et al, 2020). Their results showed that enriched radiation-sensitizing genes were involved in the nonhomologous end-joining pathway, which supports the role of the nonhomologous end-joining pathway in radiation-induced DNA damage repair, as reported previously (Mao et al, 2008; Santivasi & Xia, 2014; Huang & Zhou, 2020). Although the Durocher study used a dose of 3 Gy, our study employed a single high dose of 20 Gy and implicated a different set of genes. We identified *BCL2L1*/*MCL1* and *IL1R1*/*IL1RAP* as genes whose loss leads to radiation sensitization or resistance. We anticipate that the genes we uncovered are involved in overall outcomes after radiation but not the genes involved in immediate DNA damage repair. Indeed, we were able to confirm our results using both in vitro and in vivo experiments.

IL1R1 and IL1RAP are cell surface proteins that function as co-receptors involved in mediating the IL-1 cytokine family signaling pathway (Acuner Ozbabacan et al, 2014). IL-1α or IL-1β forms a complex with IL1R1 and IL1RAP at the cell membrane, mediating the acute phase of the proinflammatory process during infection or tissue damage, which initiates the IL-1 signaling pathway and leads to recruitment of adaptor proteins like myeloid differentiation primary response gene 88 (MYD88), Toll-interacting protein (TOLLIP), and IL-1 receptor-associated kinase 4 (IRAK4). The synthesis of IL-1 is induced by a variety of stimuli including endotoxin, other cytokines, bacterial or virus infection, and IR (Stoecklein et al, 2015). Specifically to radiation, IL1 is released by radiation damaged epithelium or stressed/necrotic cells and rapidly initiate the production of chemokines and inflammatory cytokines (Chen et al, 2007). As a matter of fact, radiation-induced IL-1 is highly relevant to radiation-induced pneumonitis and skin fibrosis (Müller & Meineke, 2007), which are the results of stimulation of proliferation of keratinocytes and fibroblasts and the induction of matrix metalloproteases and collagen synthesis (Chen et al, 2005; Lierova et al, 2018). IRAK1 is a downstream component of the IL-1/IL1R1 pathway. Recently, Liu and colleagues discovered that IRAK1 inhibitors can overcome radiation resistance in TP53-mutated cell lines, that is, head and neck squamous cell carcinoma, breast cancer, and colorectal cancer. The authors employed drug-based strategies to identify genes related to RT resistance and found that IRAK1 deletion re-sensitized HeLa cell lines to RT through PIN1 (Liu et al, 2019). However, their results are apparently different from our observation using MCF10A cells. Our screening results indicated that IRAK1 depletion may result in radiation resistance, suggesting that multiple mechanisms link the IL-1/IL1R pathway with radiation response and the outcomes may vary depending on tumor context.

At the downstream effector level, IL-1 signaling can activate two major pathways, IKK–IκB–NF-κB and/or MKK-MAPK/JNK/ERK kinase pathway. The inhibitor of NF-κB kinase subunit beta (IKKβ) is activated and phosphorylates the NF-κB inhibitor (IκB), which leads to the release of NF-κB and allows its translocation to the nucleus to activate the expression of many downstream genes (Dinarello, 2009, 2011). In our mechanistic inquiry of radiation resistance, we performed whole-genome RNA-seq to investigate the potential downstream effectors of IL1R1 and IL1RAP in radiation resistance (Fig 3A and B). We found that the NF-κB signal pathway was down-regulated in IL1R1-KO and IL1RAP-KO cells after RT, which was further verified by using NFKB1-KO and IKBKG-KO in MCF10A cells (Fig 3D and E). Moreover, our whole-genome RNA-seq data also revealed additional sets of genes (i.e., genes in clusters 1, 2, and 4) that are regulated after radiation but in an IL1R1/IL1RAP–independent manner. This information will be of interest to investigators studying transcriptional changes in response to RT.

Previous studies indicated that IR-induced activation of NF-κB in certain cancers was linked with tumor resistance to radiation (Ahmed & Li, 2008; Li & Sethi, 2010). NF-κB is recognized as a key feature in protecting cells from apoptosis across various cell types (Chen et al, 2002; Ahmed et al, 2013). Blocking NF-κB inhibited the adaptive radioresistance in murine epidermal cells (Fan et al, 2007). However, although the inhibition of NF-κB activation increases apoptotic response and decreases the growth and clonogenic survival of several human cancer cell lines, not all experiments demonstrate enhanced radiosensitivity with NF-κB inhibition. For instance, in prostate cancer cells, the inhibition of NF-κB by a negative super-repressor IκB mutant enhances apoptosis in DU145 (Flynn et al, 2003) but not in PC3 cells (Pajonk et al, 1999). These data suggest that the impact of NF-κB on cellular sensitivity to radio/chemotherapy is highly context dependent. Like wisely, our data suggest that knockout of NF-κB caused cells to resist radiation treatment (Fig 3D and E). Because the roles of NF-κB in tumor progression and/or responses to radio/chemotherapy are highly context dependent, extensive future studies are required before we can implement any strategy to target this pathway for cancer treatment.

On the other hand, our results suggest that targeting BCL2 family proteins may be a reliable way to enhance RT, especially in solid tumors. Extensive studies have already provided evidence supporting the roles of BCL2 family proteins such as Bcl2, Bcl-XL, and Mcl-1 in mediating resistance to apoptosis induced by chemotherapy or radiotherapy (Datta et al, 1995). Elevated BCL2 family proteins expression have been revealed in multiple human cancers, like gastric cancer (Park et al, 2015), ovarian cancer (Kurita et al, 2012; Xu et al, 2022), lung cancers (Haura et al, 2004), which are associated with chemoresistance, radioresistance, and poor prognosis. The ability of BCL2L1 to prevent apoptosis in response to chemotherapy-induced DNA damage and cell-cycle arrest has been implicated (Minn et al, 1995). Notably, BCL2L1 is considered the most potent member of its family inducing chemoresistance, and its expression level is regarded as an important indicator of chemotherapeutic efficacy (Cardenas et al, 2017). Therefore, an attempt to overcome radiation resistance against apoptosis by inactivating Bcl-XL is a very attractive approach for anticancer therapy.

In our whole-genome CRISPR/Cas9 screen, we uncovered both BCL2L1 and MCL1 as significant hits, suggesting that loss of these anti-apoptotic genes may show synthetic lethal interaction with RT. Indeed, we validated these results and showed that the BCL2L1 inhibitor A-1331852 and the MCL1 inhibitor S63845 enhanced RT (Fig 4A and B). In consistence with our study, Loriot and colleagues developed a novel BCL-2 and BCL2L1 inhibitor S44563 and found that it showed radiosensitization in small-cell lung cancer (Loriot et al, 2014). As both BCL2L1 and MCL1 belong to the BCL2 family, it is worth noting that A-1331852, which specifically targets Bcl-xL/BCL2L1, is a selective BH3 mimetic similar to ABT-199 (venetoclax) (Wang et al, 2020b). ABT-199 has been shown to enhance the efficacy of docetaxel in solid tumors (Leverson et al, 2015). In addition, S63845, a specific inhibitor of MCL-1, has shown promise in preclinical models of triple-negative breast cancer (Merino et al, 2017). In our study, we compared the BCL2L1 and MCL1 inhibitors with the BCL2 inhibitor ABT-199. Intriguingly, ABT199 did not significantly influence cell growth with or without radiation. However, A-1331852 induced cell death in combination with radiation (Fig 4A and B). These data are consistent with a previous study that a specific BCL2L1 inhibitor BXI-72, which does not target BCL-2 or MCL-1, overcomes acquired radioresistance in lung cancer (Park et al, 2013). The complexity of anti-cell death functions of BCL-2, MCL-1, and BCL-xL may be explained by differential reliance on these anti-apoptotic molecules, which may vary depending on cell types and/or triggers of cell death. It seems that different inhibitors targeting distinct BCL2 family proteins would show efficacy in various cancers depending on the expression of these anti-apoptotic proteins (Fig S2). Moreover, we demonstrated the efficacy of this combination therapy in suppressing tumor growth in vivo using a syngeneic model (Fig 5). Taken together, our findings suggest the potential of combining inhibitors targeting BCL2 family proteins with radiation as a strategy to enhance RT in breast and other solid cancers, which can be further tested in clinical trials.

## Materials and Methods

### Cell lines

MCF10A, MDA-MB-231, MDA-MB-468, Hs578T, and three mouse breast cancer cell lines 4T1, EMT6, EO771 were purchased from the American Type Culture Collection (ATCC). 293A, MDA-MB-231, MDA-MB-468, and EO771 cells were cultured in DMEM supplemented with 10% FCS (Sigma-Aldrich). Hs578T cells were grown in DMEM with 10% FCS and 0.01 mg/ml human insulin (Sigma-Aldrich). MCF10A normal breast epithelial cells were grown in DMEM/F12 medium supplemented with 20 ng/ml epithelial growth factor (EGF; Thermo Fisher Scientific), 100 ng/ml cholera toxin (Sigma-Aldrich), 10 μg/ml insulin (Sigma-Aldrich), 0.5 mg/ml hydrocortisone (Sigma-Aldrich), and 5% horse serum (Thermo Fisher Scientific). 4T1 cells were cultured in Roswell Park Memorial Institute 1640 medium with 10% FBS. EMT6 cells were grown in Waymouth MB 752/1 Medium with 2 mM L-glutamine and 15% FBS. All the cell lines were tested to verify that they were free of mycoplasma contamination.

### Antibodies and inhibitors

Antibodies used in this study are those for IL1RAP (73070S); IκBα, NF-κB1, and IKBKG (9936T); cleaved PARP (5625S); PARP (cat. 9542S); cleaved caspase-3 (9661S); and BCL-xL and BAX (9941T; all from Cell Signaling Technology); vinculin (V9264; Sigma-Aldrich); and GAPDH (sc-32233; Santa Cruz Biotechnology).

Inhibitor A-1331852 (HY-19741) was purchased from MedChemExpress. S63845 (21131) was purchased from Cayman Chemical. ABT-199 (S8048) was from Selleck Chemicals. IL-1α human recombinant (SRP3310) and IL-1β human recombinant (SRP3083) were purchased from Sigma-Aldrich.

### CRISPR/Cas9–based screening

The TKOv3 library contains 71,090 gRNAs, consisting of 70,948 gRNAs targeting 18,053 protein-coding genes (4 gRNAs/gene) and 142 EGFP-, LacZ-, and luciferase-targeted control gRNAs (Hart et al, 2017). This library was a gift from Dr Traver Hart (MD Anderson Cancer Center). The library generation and virus preparation were described previously (Tang et al, 2021). The screen was conducted as described before. Briefly, MCF10A cells were infected with the TKOv3 library lentivirus at at least one of ∼0.25 to maintain representation of every gRNA in at least ∼250 cells. 24 h later, cells were cultured with fresh medium containing puromycin (2 μg/ml) for selection. After selection, cells were cultured and passaged at about 200-fold coverage. At day 5, cells were treated with 20 Gy of irradiation and then divided into two groups. Cells were passaged every 3 d. At days 5, 12, and 19, 25 million cells were collected for genomic DNA extraction (QIAGEN kit). Polymerase chain reaction was performed to amplify gRNA inserts via primers harboring Illumina TruSeq adapters with i5 and i7 barcodes as described previously (Tang et al, 2021). The resulting libraries were sequenced using an Illumina HiSeq 2500 system. Model-based Analysis of Genome-wide CRISPR/Cas9 Knockout (MAGeCK) (Li et al, 2014) and DrugZ (Hart et al, 2015) analysis were conducted to calculate the difference in gRNA enrichment between the untreated group (day 5) and radiation-treated groups (days 12 and 19).

### RNA-seq and data analysis

MCF10A wild-type, *IL1R1*-KO, *IL1RAP*-KO cells (each with two biological replicates) with or without RT were collected. Total RNA was extracted (QIAGEN), and an Illumina TruSeq Stranded Total RNA Library Prep Kit was used to prepare the library after rRNA depletion. mRNA sequencing was conducted in NextSeq 550 (Illumina) to generate 75-bp paired ends. The data are publicly deposited (GSE236331).

For RNA sequencing data analysis, quality control and data filtering were preprocessed with Cutadapt software (version 1.15). STAR (version 2.5.3a) (Dobin et al, 2013) and the human reference genome (GRCh38) were used for genome mapping. Gene abundances were calculated by HTseq-count using the ENSEMBL v83 annotations. The raw read counts of retained genes, in which at least one sample >5 reads, were analyzed by DESeq2 software (Anders & Huber, 2010). *P*-values were customized by the Benjamini and Hochberg approach to control for FDR. Differentially expressed genes were defined as those with a fold change (FC) > 1.35 and FDR <0.05. Standard gene set enrichment analysis was conducted with a hypergeometric test using RDAVID Web-Service (version 1.19.0) (Fresno & Fernández, 2013).

### Radiation treatment

Cells were seeded in 10-cm dishes or six-well plates 1 d before RT, which was performed by a Precision X-RAD 320 Biological Irradiator. For a single high dose of RT, cells were irradiated at a single dose of 20 Gy or 10 Gy at one time. For conventional treatment, cells were treated at a dose of 2 Gy in one each of five consecutive days, followed by rest for 2 d, followed by another round of 2 Gy of irradiation in one each of consecutive days, which mimics a clinical radiation schedule. Inhibitors were added at indicated times with the described dose of radiation in corresponding figures. For immunoblot analysis, cells were harvested 72 h after the end of RT.

### Generation of KO cells

*IL1R1*-KO, *IL1RAP*-KO, and other KO cells used in this study were generated by using pLentiCRISPRv2 plasmids. gRNAs against target genes were designed by Synthego. Cells were transfected and selected with 2 mg/ml puromycin for 48 h after transfection. Then the pool cells were collected for further verification by immunoblotting. gRNA sequences used for the generation of KO cells are listed below:

*IL1R1*-KO1: sgRNA: CAAGCAATATCCTATTACCC.

*IL1R1*-KO2: sgRNA: TTTGTGTTGATGAATCCTGG.

*IL1RAP*-KO1: sgRNA: GCTGCGCTTGAGATCCTCAG.

*IL1RAP*-KO2: sgRNA: GTAAGGAGAAAGATGTGCTG.

*NFKB1*-KO1: sgRNA: ACAGCTGGATGTGTGACTGG.

*NFKB1*-KO2: sgRNA: ACTGGAAGCACGAATGACAG.

*IKBKG*-KO1: sgRNA: CGTCACCTGGGCTTTCACAG.

*IKBKG*-KO2: sgRNA: GAGGAGAATCAAGAGCTCCG.

*IKBKG*-KO3: sgRNA: TGGGCGAAGAGTCTCCTCTG.

### Western blot analysis

Cells were lysed in 1 × sodium dodecyl sulfate gel-loading buffer (50 mM Tris–HCl [pH 6.8], 2% sodium dodecyl sulfate, 10% glycerol, 4% β-mercaptoethanol, and 0.025% bromophenol blue) and boiled 10 min for further analysis. Samples were separated by sodium dodecyl sulfate polyacrylamide gel electrophoresis and analyzed by immunoblotting with indicated antibodies.

### Cell growth measurements

For the CellTiter-Glo Luminescent Cell Viability Assay (Promega), cells were seeded in 96-well plates with six replicates and treated as indicated. Luminescence detection was performed at indicated time points according to the standard protocol using a BioTek Synergy multimode microplate reader. For cell proliferation assays, cells were grown in 6-well or 12-well plates with indicated treatments. Cell numbers were counted with an automated cell counter (TC20; Bio-Rad) and calculated accordingly.

### Crystal violet staining and quantification assay

For the crystal violet staining and quantification assay, cells were seeded in 6-well plates or 12-well plates and treated with indicated treatments. Cells were maintained for indicated times. After the removal of media, cells were stained with crystal violet staining solution (Sigma-Aldrich) for 5–10 min at room temperature. The staining solution was then removed, and plates were gently washed by dipping into water several times and air dried overnight. For quantification, 1 ml of 10% of acetic acid was added into each well and incubated for 20 min on a shaker. A total of 100 μl of each sample was transferred to 96-well plate, and optical density was measured at a wavelength of 590 nm by a BioTek Synergy multimode microplate reader.

### Immunohistochemical staining and scoring

Slides deparaffinization, antigen retrieving, and blocking were performed according to protocols described previously (Yin et al, 2019). Anti–Ki-67 antibody was from Abcam (ab16667) and anti-cleaved caspase-3 was from was Cell Signaling Technology (9664S). Cell nuclei were stained with hematoxylin.

### Animal model

For in vivo syngeneic studies, 1 × 10^5^ 4T1 cells were subcutaneously injected into right flank of 6-wk-old female BALB/cJ mice (The Jackson Laboratory). Cells were suspended in PBS and mixed with Matrigel in a 1:1 ratio (volume) before injection. Eight mice were used per group. Mice were randomly assigned to four groups and were treated with vehicle control/PBS, radiation (a single dose of 5 Gy), inhibitor A-1331852 (406841; Medkoo), or the combination treatment. Drug treatment started as soon as the tumor became palpable. A concentration of 25 kg/mg A-1331852 was given by oral gavage daily for 2 wks except for 2 d without treatment at the end of each week. A-1331852 was dissolved in 60% Phosal 50 PG (HY-Y1903, MedChemExpress), 27.5% PEG400 (HY-Y0873A, MedChemExpress), 10% ethanol, and 2.5% dimethyl sulfoxide (Leverson et al, 2015). Radiation was administrated for 7 d when the tumor volume reached ∼100 mm^2^. The mice were anesthetized via isoflurane and positioned on a platform with a Cerrobend jig shielding the body. RT was performed using an image-guided small animal irradiator (X-RAD SmART; Precision X-Ray Inc.) with integrated cone beam computed tomography (CT; 60 kVp, 1 mA). The x-ray beam was operated at 225 kV, 20 mA. Cone beam CT images were acquired using a 2-mm aluminum filter. For radiation delivery, the beam was hardened using a 0.3-mm copper filter. A 15-mm collimator was used to deliver a dose of 5 Gy. Tumor sizes were measured by an electronic caliper twice a week starting at 6 d after injection. The tumor volume (V) was calculated by the formula: V = 1/2 × length × width^2^. Mice were euthanized by CO_2_ inhalation at the end of the experiment, and the tumors were excised for analysis. The animals were housed in pathogen-free facilities. All animal experiments were approved by the MD Anderson Cancer Center Animal Care and Use Committee.

### Statistical analysis

Data analyses were performed with unpaired *t* tests or one-way analysis of variance using GraphPad Prism software (version 8.0), unless otherwise noted. A *P*-value of <0.05 was considered statistically significant.

## Data Availability

The mRNA sequence data from this publication have been deposited to the GEO DataSets (https://www.ncbi.nlm.nih.gov/gds/) and assigned the identifier GSE236331.

## Supplementary Material

Reviewer comments
