# Supplementary material for: Genome-wide CRISPR screen reveals the synthetic lethality between BCL2L1 inhibition and radiotherapy
Source: Life Sci Alliance. 2024 Feb 5;7(4):e202302353. doi: 10.26508/lsa.202302353 (PMC10844523; doi:10.26508/lsa.202302353)
Supplement: Reviewer comments [file LSA-2023-02353_review_history.pdf]

Life Science Alliance

Genome-wide CRISPR screen reveals the synthetic lethality between BCL2L1 inhibition and radiotherapy

Ling Yin, Xiaoding hu, Guangsheng Pei, Mengfan Tang, You Zhou, Huimin Zhang, Min Huang, Siting Li, Jie Zhang, Citu Citu, Zhongming Zhao, Bisrat Debeb, Xu Feng, and Junjie Chen
DOI: https://doi.org/10.26508/lsa.202302353

Corresponding author(s): Junjie Chen, The University of Texas MD Anderson Cancer Center and Xu Feng, The University of Texas MD Anderson Cancer Center

Review Timeline:

Submission Date:	2023-09-02
Editorial Decision:	2023-11-07
Revision Received:	2023-12-09
Editorial Decision:	2024-01-19
Revision Received:	2024-01-21
Accepted:	2024-01-22

Scientific Editor: Eric Sawey, PhD

Transaction Report:

(Note: With the exception of the correction of typographical or spelling errors that could be a source of ambiguity, letters and reports are not edited. The original formatting of letters and referee reports may not be reflected in this compilation.)

November 7, 2023

Re: Life Science Alliance manuscript #LSA-2023-02353-T

Prof. Junjie Chen
The University of Texas MD Anderson Cancer Center
Experimental Radiation Oncology
1515 Holcombe Boulevard
Houston, TX 77030

Dear Dr. Chen,

Thank you for submitting your manuscript entitled "Genome-wide CRISPR screen reveals the synthetic lethality between BCL2L1 inhibition and radiotherapy" to Life Science Alliance. The manuscript was assessed by expert reviewers, whose comments are appended to this letter. We invite you to submit a revised manuscript addressing the Reviewer comments.

To upload the revised version of your manuscript, please log in to your account: https://lsa.msubmit.net/cgi-bin/main.plex

You will be guided to complete the submission of your revised manuscript and to fill in all necessary information. Please get in touch in case you do not know or remember your login name.

While you are revising your manuscript, please also attend to the below editorial points to help expedite the publication of your manuscript. Please direct any editorial questions to the journal office.

The typical timeframe for revisions is three months. Please note that papers are generally considered through only one revision cycle, so strong support from the referees on the revised version is needed for acceptance.

When submitting the revision, please include a letter addressing the reviewers' comments point by point.

We hope that the comments below will prove constructive as your work progresses.

Thank you for this interesting contribution to Life Science Alliance. We are looking forward to receiving your revised manuscript.

Sincerely,

Eric Sawey, PhD
Executive Editor
Life Science Alliance
http://www.lsajournal.org

A. THESE ITEMS ARE REQUIRED FOR REVISIONS

-- A letter addressing the reviewers' comments point by point.

-- An editable version of the final text (.DOC or .DOCX) is needed for copyediting (no PDFs).

-- High-resolution figure, supplementary figure and video files uploaded as individual files: See our detailed guidelines for preparing your production-ready images, https://www.life-science-alliance.org/authors

-- Summary blurb (enter in submission system): A short text summarizing in a single sentence the study (max. 200 characters including spaces). This text is used in conjunction with the titles of papers, hence should be informative and complementary to the title and running title. It should describe the context and significance of the findings for a general readership; it should be written in the present tense and refer to the work in the third person. Author names should not be mentioned.

-- By submitting a revision, you attest that you are aware of our payment policies found here: https://www.life-science-alliance.org/copyright-license-fee

B. MANUSCRIPT ORGANIZATION AND FORMATTING:

Full guidelines are available on our Instructions for Authors page, https://www.life-science-alliance.org/authors

We encourage our authors to provide original source data, particularly uncropped/-processed electrophoretic blots and spreadsheets for the main figures of the manuscript. If you would like to add source data, we would welcome one PDF/Excel-file per figure for this information. These files will be linked online as supplementary "Source Data" files.

IMPORTANT: It is Life Science Alliance policy that if requested, original data images must be made available. Failure to provide original images upon request will result in unavoidable delays in publication. Please ensure that you have access to all original microscopy and blot data images before submitting your revision.

Reviewer #1 (Comments to the Authors (Required)):

This is a nice study by Yin et al. where a whole genome CRISPR KO screening has been done in non-malignant human beast epithelial cells (MACF10A) has been done to find out to potential explore the genes/pathways which play important role in radiation resistance. The data suggests that loss of IL-R1pathway led to radiation resistance, while anti-apoptotic member BCL2L1 is crucial to protect cell death against radiation. The finding is novel but lack of appropriate approach impacts the importance of the study. Here are my major concerns.
• Why non-malignant human beast epithelial cells (MACF10A) was chosen for whole genome CRISPR KO screening, why not a human breast cancer line (any subtype of interest? It is expected that non-malignant cells and malignant cells would respond differently to radiation therapy. In this study, the targets were selected from a non-malignant cells (MCF10A) and therapeutic efficacy was tested in malignant cells (MDA-MB-231, MDA-MB-468, 4T1 and many more).
• From RNA sequencing data, what is the rationale behind choosing cluster 3? Why not cluster 1, where the enrichment ration is higher. There is possibility that these pathways and associated gene might have better relevance to radiation response as compared to cluster 1.
• The in vivo studies has been done in a mouse cancer lines, which limits its translational relevance, it should have been done in human breast cancer line. I am wondering why the authors have mentioned it is a xenograft tumor? The 4T1 us a mouse breast cancer lines (derived from mammary gland tissue of BALB/c) and tumors were developed in BALB/c mice in Figure 5. I thought it should be mentioned as Syngeneic tumor model.

Reviewer #2 (Comments to the Authors (Required)):

The manuscript submitted by Chun et al is quite complicated and is not presented in intelligent manner.
Author uses many reagents but without careful thinking. One of the major weakness of the study is that that frequently changes cell line in experiments without logic and keep on describing results
Most of the information which authors have in their manuscript is already known so from that point of view, the study is not entirely noval
I would give one examples , in figure 4 , why author did not include MCF10A cells are not included in IR+ inhibitors groups. Why authors only selected 4T1 cells for in vivo experiments in not clear
Figure 3 E is there but no corresponding description is there in the text

Overall , the manuscript need severe re drafting and re analysis before this paper is readable
It is also not clear why author did not generate ILR1/RAP mutants in all cell line tested

Reviewer #3 (Comments to the Authors (Required)):

Precise mechanism underlying radiation-induced cell killing, although extensively studied, remains unevaluated. In this study, a whole-genome CRISPR loss-of-function screen was conducted which demonstrates a gene pattern illustrating elements of both temporal inherent and acquired responses to RT. Using this unique approach, IL1R1 (interleukin 1 receptor, type I), an interleukin receptor is identified to be responsible for the acquired radioresistance and is upregulated by the well-defined stress responsive NF-κB pathway. In the screen list, as expected, the mitochondrial anti-apoptotic pathway, particularly the BCL2L1 gene, is indicated in the radioresistant potential. Blocking BCL2L1 with RT demonstrated radiosensitizing effects in an array of breast cancer cells and syngeneic breast tumors. This work provides some innovative insights of cell response to radiation by whole-genomic screen and a potential target to radiosensitize breast cancer cells. Some weaknesses are raised on the screening of normal breast epithelial cells although the later data support the result using breast cancer cells.

Scientific Comments:

• The scale of the Introduction needs to be expanded to cover the data of tumor-acquired radioresistance in addition to the apoptotic pathways since apoptotic cell death is a type of acute cell death that is shown to be induced mins to hours after radiation whereas acquired radioresistance is linked to chronicle events including stem cell repopulation with DNA repair

enhancement, adaptive metabolic rewiring, and tumor clonal evolution.

• A graphic cartoon may be considered to show the pathway of lack of IL1R1 induced radioresistance.

• MCF10A is more radiosensitive compared to BC cell lines. In Fig. 1, it is unclear why normal rather than malignant cells (or radioresistant BC lines) were applied in genome CRISPR screens. Established radioresistant BC lines could be a better choice for identifying the gene responsible for the acquired gene in radioresistant BC cells and/or tumors. Results showing radioresistant BC cells with reduced or lack of L1R1/IL1RAP will add support to the conclusion. Or database analysis of L1R1/IL1RAP in recurrent/metastatic tumor v primary biopsy without IR would be appreciated.

• In Fig. 1(D) and (E), Overlay of the genes whose depletion conferred resistance to radiation between the early and late periods.(E) Overlay of the genes whose loss led to radiation sensitization between the early and late periods. This part is unclear. please explain what the early and late periods are.

• Fig. 2. KO of IL1R1 or IL1RAP leads to radiation resistance in MCF-10A cells indicating that lack of IL response is associated with cellular radiosensitivity which hold a potential new mechanism of radiation-induced cytokines probably via self and/or paracrine pathway which should be discussed.

• Fig. 3 shows downstream events of lacking IL1R of NF-κB activation which is well defined in radioresistance. A missing gap seems to be how the lack of IL1R leads to NF-κB activation. Is it possible that IL1R is required for inhibiting NF-kB, or IL1R is required to enhance the inhibitory partner of NF-kB?

• This work identified BCL2L1 as a new target for BC radiosensitization although it is well-demonstrated that pro-apoptosis can enhance tumor radiosensitization. The unique feature of BCL2L1 in radiation-induced apoptotic response needs to be discussed.

Response to reviewers

Reviewer #1 (Comments to the Authors (Required)):

This is a nice study by Yin et al. where a whole genome CRISPR KO screening has been done in non-malignant human beast epithelial cells (MACF10A) to potential explore the genes/pathways which play important role in radiation resistance. The data suggests that loss of IL-R1 pathway led to radiation resistance, while anti-apoptotic member BCL2L1 is crucial to protect cell death against radiation. The finding is novel but lack of appropriate approach impacts the importance of the study. Here are my major concerns.

Thank you for the nice summary and suggestions that help us improving our manuscript. We have now addressed the concerns you raised. Please see below for details.

• Why non-malignant human beast epithelial cells (MACF10A) was chosen for whole genome CRISPR KO screening, why not a human breast cancer line (any subtype of interest)? It is expected that non-malignant cells and malignant cells would respond differently to radiation therapy. In this study, the targets were selected from a non-malignant cells (MCF10A) and therapeutic efficacy was tested in malignant cells (MDA-MB-231, MDA-MB-468, 4T1 and many more).

We agree with the reviewer that non-malignant cells and malignant cells could respond differently to radiation therapy. The reason we chose a mammary epithelial cell line MCF10A rather than a human breast cancer cell line is that normal cells may not exhibit as much heterogeneity and genetics alternations as tumor cell lines, which allows us to explore all possible genetic determinants of cellular response to radiation therapy. Additionally, regardless the cell line we chose for screening, subsequent validation experiments using the same and additional cell lines are needed to confirm our conclusions. As we showed in this study, we verified our screen results not only in MCF10A cells, but also in multiply human and murine breast cancer cell lines.

• From RNA sequencing data, what is the rationale behind choosing cluster 3? Why not cluster 1, where the enrichment ration is higher. There is possibility that these pathways and associated gene might have better relevance to radiation response as compared to cluster 1.

We chose cluster 3 but not other clusters since these experiments were designed to investigate the potential mechanisms underlying the roles of IL1R1 and IL1RAP in radiation resistance. Thus, we conducted whole genome RNA-seq analysis using wild-type cells as well as IL1R1-KO and IL1RAP-KO cells. Clustering analysis indicate that Cluster 1, Cluster 2 as well as Cluster 4 are genes changed in all cells regardless of their genotypes (i.e. WT, IL1R1-KO, and IL1RAP-KO) following RT treatment when compared with control cells without RT treatment (**Figure 3A**). Genes from cluster 1 and cluster 2 were upregulated following RT, while genes in cluster 4 were downregulated in cells treated with RT.

As mentioned above, we were particularly interested in the mechanisms underlying radiation resistance observed in IL1R1-KO and IL1RAP-KO cells. Indeed, genes presented in Cluster 3 were significantly downregulated in IL1R1-KO and IL1RAP-KO cells when compared with those in wild-type cells. These data indicate that radiation treatment led to transcriptional upregulation of a set of genes in an IL1R1- and IL1RAP-dependent manner, which accounts for, at least in part, IL1R1/IL1RAP-mediated radiation sensitivity.

We agree with this reviewer that the genes identified in Clusters 1, 2, and 4 are also potentially interesting, since the expression of these genes were changed after radiation. These data are presented in our manuscript, which will be of interest to any investigators who would like to further pursue radiation-induced events.

• The in vivo studies have been done in a mouse cancer lines, which limits its translational relevance, it should have been done in human breast cancer line. I am wondering why the authors have mentioned it is a xenograft tumor? The 4T1 is a mouse breast cancer line (derived from mammary gland tissue of BALB/c) and tumors were developed in BALB/c mice in Figure 5. I thought it should be mentioned as Syngeneic tumor model.

The reviewer is correct by pointing out 4T1 mouse model as syngeneic tumor model rather than xenograft tumor model. We correct it in the revised manuscript.

We agree with this reviewer the potential limitation of our study, since the combination therapy of BCL2L1 inhibitor and irradiation treatment was conducted using a murine mammary cancer cell line 4T1. Nevertheless, we did verify BCL2L1 inhibitor has a synergistic effect with RT not only in MCF10A cells (**Figure 4A**), but also in multiple human breast cancer cells (MDA-MB-231, MDA-MB-468, and Hs578T) and various murine breast cancer cells (4T1, EMT6, and EO771) *in vitro* (**Figure 4B**). For animal experiments, we chose the murine syngeneic model 4T1 to validate the effect of this combination therapy on solid tumors. This model, being immunocompetent, may be broadly analogous to that of human patients, since increasing evidence suggest that immune responses also contribute to the efficacy of radiation treatment in humans. However, we did not evaluate the contribution of immune responses in this paper, which will be explored in future studies.

Reviewer #2 (Comments to the Authors (Required)):

The manuscript submitted by Chun et al is quite complicated and is not presented in intelligent manner. Author uses many reagents but without careful thinking. One of the major weaknesses of the study is that that frequently changes cell line in experiments without logic and keep on describing results.

Thank you for your insights and valuable suggestions on our data presentation.

Firstly, we performed the screen using a mammary epithelial cell line MCF10A with a single dose (20 Gy) of RT. DrugZ analysis revealed that genes whose loss confer radiation resistance are mainly genes involved in IL1R1/IL1RAP-dependent pathway, indicating that this pathway plays an important role in radiation resistance. Additionally, genes whose loss lead to radiation sensitization are anti-apoptotic genes including BCL2L1. Secondly, we verified our screening results using IL1R1-KO and IL1RAP-KO cells and performed RNA-seq to reveal that the underlying mechanisms likely involve NF-κB signal pathway and transcriptional regulation following RT. Thirdly, we verified that BCL2L1 inhibitor could be used effectively in combination with RT in MCF10A cells as well as in multiply human breast cancer cells (MDA-MB-231, MDA-MB-468, and Hs578T) and various murine breast cancer cells (4T1, EMT6, and EO771), suggesting that this class of inhibitors may be used to enhance radiation therapy. Finally, we used syngeneic 4T1 murine model and further validated the effect of this combination therapy on solid tumors in an immunocompetent microenvironment. Together, our data not only revealed key pathways involved in radiation sensitization and resistance, but also highlight the potential of combining BCL2L1 and/or other BCL2 family inhibitors with radiation to enhance the efficacy of current radiation therapy.

As mentioned above in our response to Reviewer #1, we chose MCF10A cells since these non-tumorigenic cells may allow us to explore all possible genetic determinants of cellular response to radiation. We further validated our results using additional KO cells as well as in multiple cell lines. Furthermore, we used not only human but also murine cancer cell lines to validate the effect of BCL2L1 inhibitor in combination with radiation. Our results using multiple cell lines confirmed the potential of combination BCL2L1 inhibitor with radiation for the treatment of solid tumors such as breast cancers.

• Most of the information which authors have in their manuscript is already known so from that point of view, the study is not entirely novel.

We agree with the reviewer that our study is not entirely novel. Nevertheless, our study provides additional information that are useful to investigators who are interested in radiation therapy.

For example, in our mechanistic inquiry of radiation resistance, we performed whole genome RNA-seq to investigate the potential downstream effectors of IL1R1 and IL1RAP in radiation resistance. We found that NF-κB signal pathway was downregulated in IL1R1-KO and IL1RAP-KO

cells following RT, which was further verified in MCF10A NFKB1-KO and IKBKG-KO cells. Moreover, these whole genome RNA-seq data will be of interest to investigators studying transcriptional changes in response to RT.

Numerous studies indicated that IR induced activation of nuclear factor kappa B (NF-κB) in certain cancers was linked with tumor resistance to radiation. NF-κB is recognized as a key feature in protecting cells from apoptosis across various cell types. Blocking NF- κB inhibited the adaptive radioresistance in murine epidermal cells. However, while the inhibition of NF-κB activation increases apoptotic response and decreases the growth and clonogenic survival of several human cancer cell lines, not all experiments demonstrate enhanced radiosensitivity with NF-κB inhibition. For instance, in prostate cancer cells, the inhibition of NF-κB by a negative super-repressor IκB mutant enhances apoptosis in DU145 but not in PC3 cells. These data suggest that the impact of NF-κB on cellular sensitivity to radio/chemotherapy is highly context dependent. Likewise, our data suggests that knock out of NF-κB caused radiation resistance (Figure 3D&E). Since the roles of NF-κB in tumor progression and/or responses to radio/chemotherapy are highly context dependent, extensive future studies are required before we can implement any strategy to target this pathway in cancer treatment.

In radiation sensitization experiments, we validated that BCL2L1 inhibitor has a synergistic effect with RT not only in MCF10A cells (Figure 4A & B), but also in multiple human breast cancer cells (MDA-MB-231, MDA-MB-468, and Hs578T) and various murine breast cancer cells (4T1, EMT6, and EO771) *in vitro* (Figure 4B). Furthermore, we confirmed this combination therapy in a murine syngeneic model (Figure 5A&B). We examined the activation of the apoptosis pathway following radiation and verified that the BCL2L1 inhibitor promotes radiation sensitivity by inducing apoptosis (Figure 4D). In consistent with our study, Loriot and colleagues developed a novel BCL-2 and BCL2L1 inhibitor S44563 and found that it acts as radiosensitizer in small-cell lung cancer. Another previous study indicated that a specific BCL2L1 inhibitor BXI-72, not BCL-2 or MCL-1 inhibitors, overcomes acquired radioresistance in lung cancer. The precise effects of BCL-2, BCL-xL and/or MCL-1 inhibition may reflect cell types, cell death triggers and/or the relative dependence of these overlapping anti-apoptotic molecules/pathways. We have now included these references and discussions in the revised manuscript.

We do not fully understand the underlying mechanisms that account for the differences observed in this manuscript and some of the previous studies. Therefore, we have now included the above and additional discussions in the revised manuscript to point out these differences. Nevertheless, the publication of this manuscript will be of interest to many investigators in the field, as it allows them to carefully and critically analyze their own data, which often are derived from the use of limited cancer cell lines.

• I would give one examples, in figure 4, why author did not include MCF10A cells are not included in IR+ inhibitors groups.

Figure 4A shows MCF10A cells treated with a single high dose 20 Gy irradiation together with inhibitors A-1331852 or S63845. These results indicated that inhibiting BCL2L1 and/or MCL1 increased radiation sensitivity, which agrees with our screening results.

Additionally, we utilized A-1331852, S63845, and the BCL2 inhibitor ABT-199 in three other human breast cancer cell lines (MDA-MB-231, MDA-MB-468, and Hs578T) and murine breast cancer cells (4T1, EMT6, and EO771) treated with IR to further verify the combination therapy of BCL2 family inhibitors with RT (Figure 4B). These data support that these inhibitors may be used to enhance the efficacy of radiation therapy in solid tumors.

• Why authors only selected 4T1 cells for in vivo experiments is not clear.

Thanks for your question. After we verified that BCL2L1 inhibitor has a synergistic effect with RT not only in MCF10A cells (Figure 4A), but also in multiple human breast cancer cells (MDA-MB-231, MDA-MB-468, and Hs578T) and various murine breast cancer cells (4T1, EMT6, and EO771) *in vitro* (Figure 4B), we would like to further test this hypothesis *in vivo*. We chose the murine syngeneic model 4T1 to validate the effect of this combination therapy, since this model is widely used in the field. Additionally, the syngeneic model has intact immune responses, which is analogous to that of human patients. This is especially relevant since increasing studies indicate that the effect of radiation therapy may at least in part due to host immune responses. As suggested by this reviewer, we have now provided additional explanation in the revised manuscript.

•Figure 3 E is there but no corresponding description is there in the text.

Figure 3E showed that BCL2L1 inhibitor A-1331852 effectively reversed the radiation resistance observed in NFKB1-KO and IKBKG-KO cells (Figure 3D&E). As these results indicate that the BCL2L1 inhibitor may also be effective when used to treat cells displaying radiation resistance, we included them in the results part of "BCL2L1 inhibitor sensitizes cells to radiotherapy."

• Overall, the manuscript needs severe re drafting and re analysis before this paper is readable. It is also not clear why author did not generate ILR1/RAP mutants in all cell line tested.

Thanks for your suggestion! We have revised the manuscript accordingly to help the readers understand our rationale, results and conclusions.

We did not further pursue ILR1/RAP pathway in all cell lines tested due to two reasons. First, an early paper indicated that inhibition of this pathway led to radiation sensitization in several TP53 mutant cancer cell lines, i.e. head and neck squamous cell carcinoma, breast cancer and colorectal cancer, which is apparently different from our observations using MCF10A cells. Second, IR would stimulate various cytokines, including IL-1, which are released by radiation damaged epithelium or stressed/necrotic cells, and rapidly initiate the production of chemokines and inflammatory cytokines. Radiation-induced IL-1 is highly relevant to radiation-induced pneumonitis and skin fibrosis, which are the results of stimulation of proliferation of

keratinocytes and fibroblasts and the induction of matrix metalloproteases (MMP) and collagen synthesis. IL-1 signaling can activate two major downstream pathways, IKK–IκB–NF-κB and/or MKK-MAPK/JNK/ERK kinase pathways. We found that NF-κB signal pathway was downregulated in IL1R1-KO and IL1RAP-KO cells following RT, which we further verified using NFKB1-KO and IKBKG-KO in MCF10A cells (Figure 3D&E). However, as we discussed above, the impact of its downstream NF-κB on cellular sensitivity to radio/chemotherapy is highly context dependent. Extensive future studies are needed to fully understand the mechanisms underlying the context-dependent functions of this pathway following radiation. Thus, as for now it is unlikely that this pathway can be targeted to enhance radiation therapy. In this manuscript, we decided to further investigate BCL2 family inhibitors, since they are available in the clinic. We hope that our manuscript provides a reasonable justification for further testing the efficacy of combining these inhibitors with radiation therapy for the treatment of solid cancers including breast cancer.

Reviewer #3 (Comments to the Authors (Required)):

Precise mechanism underlying radiation-induced cell killing, although extensively studied, remains unevaluated. In this study, a whole-genome CRISPR loss-of-function screen was conducted which demonstrates a gene pattern illustrating elements of both temporal inherent and acquired responses to RT. Using this unique approach, IL1R1 (interleukin 1 receptor, type I), an interleukin receptor is identified to be responsible for the acquired radioresistance and is upregulated by the well-defined stress responsive NF-κB pathway. In the screen list, as expected, the mitochondrial anti-apoptotic pathway, particularly the BCL2L1 gene, is indicated in the radioresistant potential. Blocking BCL2L1 with RT demonstrated radiosensitizing effects in an array of breast cancer cells and syngeneic breast tumors. This work provides some innovative insights of cell response to radiation by whole-genomic screen and a potential target to radiosensitize breast cancer cells. Some weaknesses are raised on the screening of normal breast epithelial cells although the later data support the result using breast cancer cells.

Thank you for the nice summary and valuable suggestion for our manuscript.

Scientific Comments:

• The scale of the Introduction needs to be expanded to cover the data of tumor-acquired radioresistance in addition to the apoptotic pathways since apoptotic cell death is a type of acute cell death that is shown to be induced mins to hours after radiation whereas acquired radioresistance is linked to chronicle events including stem cell repopulation with DNA repair enhancement, adaptive metabolic rewiring, and tumor clonal evolution.

Thanks for your suggestion. We will expand the Introduction as suggested/highlighted above.

• A graphic cartoon may be considered to show the pathway of lack of IL1R1 induced radioresistance.

Thanks for your suggestion! We have now presented a graphic cartoon to describe the pathway involved in IL1R1/IL1RAP induced radioresistance and BCL2L1 induced radiosensitivity as Figure S2 in the revised manuscript.

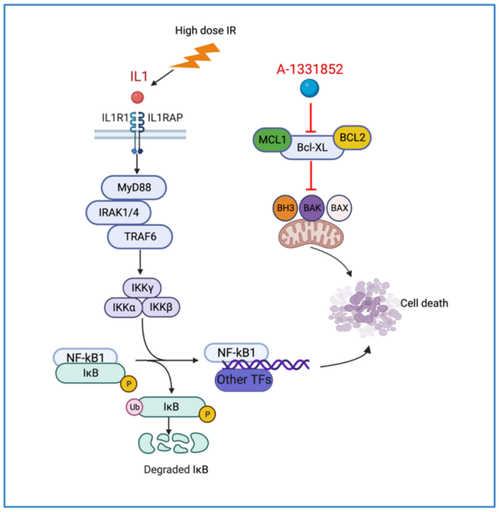

Figure S2. A proposed model of cellular response to radiation based on our screening data. Knockout of IL1RA/IL1RAP inhibits the NF-kB pathway, leading to altered transcriptional programs and the induction of radiation resistance. Additionally, the BCL2L1 inhibitor A-1331852 induces radiation sensitivity through the activation of apoptosis.

• MCF10A is more radiosensitive compared to BC cell lines. In Fig. 1, it is unclear why normal rather than malignant cells (or radioresistant BC lines) were applied in genome CRISPR screens. Established radioresistant BC lines could be a better choice for identifying the gene responsible for the acquired gene in radioresistant BC cells and/or tumors. Results showing radioresistant BC cells with reduced or lack of L1R1/IL1RAP will add support to the conclusion. Or database analysis of L1R1/IL1RAP in recurrent/metastatic tumor v primary biopsy without IR would be appreciated.

Thanks for the comments. The other two reviewers have similar concerns. The reason we chose a mammary epithelial cell line MCF10A rather than a human breast cancer cell line is that normal cells may not exhibit as much heterogeneity and genetics alternations as tumor cell lines, which allows us to explore all possible genetic determinants of cellular response to radiation therapy. Additionally, regardless the cell line we chose for screening, subsequent validation experiments using the same and additional cell lines are needed to confirm our conclusions. As we showed in this study, we verified our screen results not only in MCF10A cells, but also in multiply human and mice breast cancer cell lines.

We did not further pursue ILR1/RAP pathway in all cell lines tested due to two reasons. First, an early paper indicated that inhibition of this pathway led to radiation sensitization in several TP53 mutant cancer cell lines, i.e. head and neck squamous cell carcinoma, breast cancer and colorectal cancer, which is apparently different from our observations using MCF10A cells. Second, IR would stimulate various cytokines, including IL-1, which are released by radiation damaged epithelium or stressed/necrotic cells and rapidly initiates the production of chemokines and inflammatory cytokines. Radiation-induced IL-1 is highly relevant to radiation pneumonitis and skin fibrosis, which are the results of stimulation of proliferation of keratinocytes and fibroblasts and the induction of matrix metalloproteases (MMP) and collagen synthesis. IL-1 signaling can activate two major downstream pathways, IKK–IκB–NF-κB and/or MKK-MAPK/JNK/ERK kinase pathways. We found that NF-κB signal pathway was downregulated in IL1R1-KO and IL1RAP-KO cells following RT, which we further verified using NFKB1-KO and IKBKG-KO in MCF10A cells (Figure 3D&E). However, as we discussed above, the impact of its downstream NF-κB on cellular sensitivity to radio/chemotherapy is highly context dependent. Extensive future studies are needed to fully understand the mechanisms underlying the context-dependent functions of this pathway following radiation. Thus, as for now it is unlikely that this pathway can be targeted to enhance radiation therapy. In this manuscript, we decided to further investigate BCL2 family inhibitors, since they are available in the clinic. We hope that our manuscript provides a reasonable justification for further testing the efficacy of combining these inhibitors with radiation therapy for the treatment of solid cancers including breast cancer.

Thank you for your valuable suggestion on database analysis. Indeed, database analysis of IL1R1/IL1RAP in recurrent/metastatic tumors versus primary biopsies with or without ionizing radiation (IR) would be valuable to further test our hypothesis. However, obtaining human recurrent/metastatic tumor biopsies is comparatively rare in comparison to primary tumor biopsies. Unfortunately, after thorough searching, we did not identify sufficient such cases in public databases for this analysis. Nevertheless, this is a direction for our future studies, i.e. testing our working hypothesis using clinical data.

• In Fig. 1(D) and (E), Overlay of the genes whose depletion conferred resistance to radiation between the early and late periods. (E) Overlay of the genes whose loss led to radiation sensitization between the early and late periods. This part is unclear. please explain what the early and late periods are.

Sorry for the confusion! To gain a deeper understanding of genes associated with radiation, we collected cells at two distinct time points: one week after radiotherapy (RT) at day 12, defined as the early period, and two weeks after RT at day 19, considered as the late period. We conducted a comparative analysis between day 12 (early after treatment) and day 19 (late) with the baseline data before radiation at day 5.

• Fig. 2. KO of IL1R1 or IL1RAP leads to radiation resistance in MCF-10A cells indicating that lack of IL response is associated with cellular radiosensitivity which hold a potential new mechanism of radiation-induced cytokines probably via self and/or paracrine pathway which should be discussed.

Thanks for your advice. We have now included this discussion in the revised manuscript.

• Fig. 3 shows downstream events of lacking IL1R of NF-κB activation which is well defined in radioresistance. A missing gap seems to be how the lack of IL1R leads to NF-κB activation. Is it possible that IL1R is required for inhibiting NF-kB, or IL1R is required to enhance the inhibitory partner of NF-kB?

IL-1α or IL-1β forms a complex with IL1R1 and IL1RAP at the cell membrane, which initiates the IL-1 signaling pathway and leads to recruitment of adaptor proteins like IL-1 receptor-associated kinase 4 (IRAK4). At the downstream effector level, IL-1 signaling can activate two major pathways, IKK–IκB–NF-kB and/or MKK-MAPK/JNK/ERK kinase pathways. The inhibitor of nuclear factor kappa-B kinase subunit beta (IKKβ) is activated and phosphorylates the nuclear factor kappa-B inhibitor (IκB), which leads to the release of NF-κB and allows its translocation to the nucleus to activate the expression of many downstream genes. In other words, disruption of IL1/ILR1/IL1RAP complex will inactive NF-kB signaling, consequently inhibiting its many downstream genes.

Early studies indicated IR induced activation of transcription factor nuclear factor kappa B (NF-κB) in certain cancers linked with tumor resistance to radiation [1, 2]. NF-κB is recognized as a key feature in protecting cells from apoptosis across various cell types [3, 4]. Blocking NF- κB

inhibited the adaptive radioresistance in mouse epidermal cells [5]. However, while the inhibition of NF-κB activation increases apoptotic response and decreases the growth and clonogenic survival of several human cancer cell lines, not all experiments demonstrate enhanced radiosensitivity with NF-κB inhibition. For instance, in prostate cancer cells, the inhibition of NF-κB by a negative super-repressor IκB mutant enhances apoptosis in DU145 but not in PC3 cells [6]. These data suggest that the impact of NF-κB on cellular sensitivity to radio/chemotherapy is highly context dependent. Likewise, our data suggests that knock out of NF-κB caused radiation resistance (Figure 3D&E). Since the roles of NF-κB in tumor progression and/or responses to radio/chemotherapy are highly context dependent, extensive future studies are required before we can implement any strategy to target this pathway in cancer treatment. We have now included these and additional discussions in the revised manuscript.

To investigate the potential mechanisms underlying the roles of IL1R1 and IL1RAP in radiation resistance, we conducted whole genome RNA-seq analysis using wild-type cells as well as IL1R1-KO and IL1RAP-KO cells. Clustering analysis of gene expression profiles revealed that genes belonging to cluster 3 were significantly downregulated in IL1R1-KO and IL1RAP-KO cells following RT (Figure 3A). KEGG pathway annotations highlighted five pathways predominantly enriched in cluster 3, which may contribute to radiation resistance in these KO cells (Figure 3B and Supplementary Table 4). These enriched pathways are all related to NF-κB pathway, which suggests that downregualtion of NF-κB pathway may be at least one of the mechanisms by which loss of IL1R1 or IL1RAP1 leads to radiation resistance. To test this hypothesis, we knocked out IKBKG and NFKB1 in MCF10A cells (Figure 3C). Indeed, both NFKB1-KO and IKBKG-KO cells exhibited significant radiation resistance when compared to wild-type cells (Figure 3D&E). Moreover, we identified many components of this pathway in our screen (Figure 1D), including not only IL1R1/IL1RAP and IKBKG/NF-kB, but also MYD88 and IRAK1/4, which are known to be involved in the signaling pathway from IL1R1/IL1RAP to NF-kB. These data together support that KO of this pathway leads to radiation resistance in MCF10A cells.

• This work identified BCL2L1 as a new target for BC radiosensitization although it is well-demonstrated that pro-apoptosis can enhance tumor radiosensitization. The unique feature of BCL2L1 in radiation-induced apoptotic response needs to be discussed.

Thanks for your advice. We have now added this discussion as suggested.

January 19, 2024

RE: Life Science Alliance Manuscript #LSA-2023-02353-TR

Prof. Junjie Chen
The University of Texas MD Anderson Cancer Center
Experimental Radiation Oncology
1515 Holcombe Blvd.
Houston, TX 77030

Dear Dr. Chen,

Thank you for submitting your revised manuscript entitled "Genome-wide CRISPR screen reveals the synthetic lethality between BCL2L1 inhibition and radiotherapy". We would be happy to publish your paper in Life Science Alliance pending final revisions necessary to meet our formatting guidelines.

Along with points mentioned below, please tend to the following:
-please address Reviewer 3's 2nd point
-please be sure that the authorship listing and order is correct
-please upload all figure files as individual ones, including the supplementary figure files; all figure legends should only appear in the main manuscript file
-please add your main, supplementary figure, and table legends to the main manuscript text after the references section
-please add ORCID ID for the secondary corresponding author--they should have received instructions on how to do so
-please add the Twitter handle of your host institute/organization as well as your own or/and one of the authors in our system
-please add callouts for Figures S1A-C and S3 to your main manuscript text

Figure Checks:
-please add sizes next to all blots

If you are planning a press release on your work, please inform us immediately to allow informing our production team and scheduling a release date.

LSA now encourages authors to provide a 30-60 second video where the study is briefly explained. We will use these videos on social media to promote the published paper and the presenting author (for examples, see https://twitter.com/LSAjournal/timelines/1437405065917124608). Corresponding or first-authors are welcome to submit the video. Please submit only one video per manuscript. The video can be emailed to contact@life-science-alliance.org

To upload the final version of your manuscript, please log in to your account: https://lsa.msubmit.net/cgi-bin/main.plex
You will be guided to complete the submission of your revised manuscript and to fill in all necessary information. Please get in touch in case you do not know or remember your login name.

To avoid unnecessary delays in the acceptance and publication of your paper, please read the following information carefully.

A. FINAL FILES:

These items are required for acceptance.

-- An editable version of the final text (.DOC or .DOCX) is needed for copyediting (no PDFs).

-- High-resolution figure, supplementary figure and video files uploaded as individual files: See our detailed guidelines for preparing your production-ready images, https://www.life-science-alliance.org/authors

-- Summary blurb (enter in submission system): A short text summarizing in a single sentence the study (max. 200 characters including spaces). This text is used in conjunction with the titles of papers, hence should be informative and complementary to the title. It should describe the context and significance of the findings for a general readership; it should be written in the present tense and refer to the work in the third person. Author names should not be mentioned.

B. MANUSCRIPT ORGANIZATION AND FORMATTING:

Full guidelines are available on our Instructions for Authors page, https://www.life-science-alliance.org/authors

We encourage our authors to provide original source data, particularly uncropped/-processed electrophoretic blots and spreadsheets for the main figures of the manuscript. If you would like to add source data, we would welcome one PDF/Excel-file per figure for this information. These files will be linked online as supplementary "Source Data" files.

Submission of a paper that does not conform to Life Science Alliance guidelines will delay the acceptance of your manuscript.

It is Life Science Alliance policy that if requested, original data images must be made available to the editors. Failure to provide original images upon request will result in unavoidable delays in publication. Please ensure that you have access to all original data images prior to final submission.

The license to publish form must be signed before your manuscript can be sent to production. A link to the electronic license to publish form will be available to the corresponding author only. Please take a moment to check your funder requirements.

Reviews, decision letters, and point-by-point responses associated with peer-review at Life Science Alliance will be published online, alongside the manuscript. If you do want to opt out of having the reviewer reports and your point-by-point responses displayed, please let us know immediately.

Thank you for your attention to these final processing requirements. Please revise and format the manuscript and upload materials within 7 days.

Thank you for this interesting contribution, we look forward to publishing your paper in Life Science Alliance.

Sincerely,

Eric Sawey, PhD
Executive Editor
Life Science Alliance
http://www.lsajournal.org

Reviewer #1 (Comments to the Authors (Required)):

For transnational relevance, the combinatorial in vivo efficacy in figure 5 should be evaluated in xenograft using human breast cancer lines. Checking in mouse tumor cells reduces the impact of the study

Reviewer #2 (Comments to the Authors (Required)):

The manuscript is acceptable in current format , however minor language correction is required which can be done at the time of proof.

Reviewer #3 (Comments to the Authors (Required)):

The authors have improved the manuscript with proper explanations of some inquiries including the major point of using MCF-10A cells. Since the MCF-10A cells were the original resource of data generated which has led to confusion of cancer cell data being generated, the paper could be further improved to achieve an acceptable version by considering the following points:

1. The title should be modified to delete "Genome-wide CRISPR screen".
2. In the Fig 1 result and discussion, the explanation for using MCF-10A cell data resource may be added.

Response to the reviewers

Reviewer #1 (Comments to the Authors (Required)):

For translational relevance, the combinatorial in vivo efficacy in figure 5 should be evaluated in xenograft using human breast cancer lines. Checking in mouse tumor cells reduces the impact of the study.

We agree with this reviewer that the inclusion of human breast cancer xenograft models would add to the potential translational relevance of this study. As we mentioned in our manuscript, increasing evidence suggest that immune responses also contribute to the efficacy of radiation treatment in humans. Thus, we used the murine syngeneic model 4T1 to confirm the efficacy of this combination treatment. Nevertheless, we verified that BCL2L1 inhibitor has a synergistic effect with RT in multiple human breast cancer cells (MDA-MB-231, MDA-MB-468, and Hs578T). Future studies especially clinical trials are needed to further test the efficacy of this combination therapy.

Reviewer #2 (Comments to the Authors (Required)):

The manuscript is acceptable in current format, however minor language correction is required which can be done at the time of proof.

Thank you for your support!

Reviewer #3 (Comments to the Authors (Required)):

The authors have improved the manuscript with proper explanations of some inquiries including the major point of using MCF-10A cells. Since the MCF-10A cells were the original resource of data generated which has led to confusion of cancer cell data being generated, the paper could be further improved to achieve an acceptable version by considering the following points:

Thanks!

1. The title should be modified to delete "Genome-wide CRISPR screen".

Thanks for your suggestion. We wish to keep “Genome-wide CRISPR screen” in the title, since it indicates that our study was built on an unbiased screen, which adds to the significance of our manuscript.

2. In the Fig 1 result and discussion, the explanation for using MCF-10A cell data resource may be added.

Thanks! As suggested, we added the explanation for the use of MCF-10A cells in figure 1 result section and discussion section.

January 22, 2024

RE: Life Science Alliance Manuscript #LSA-2023-02353-TRR

Prof. Junjie Chen
The University of Texas MD Anderson Cancer Center
Experimental Radiation Oncology
1515 Holcombe Blvd.
Houston, TX 77030

Dear Dr. Chen,

Thank you for submitting your Research Article entitled "Genome-wide CRISPR screen reveals the synthetic lethality between BCL2L1 inhibition and radiotherapy". It is a pleasure to let you know that your manuscript is now accepted for publication in Life Science Alliance. Congratulations on this interesting work.

The final published version of your manuscript will be deposited by us to PubMed Central upon online publication.

Your manuscript will now progress through copyediting and proofing. It is journal policy that authors provide original data upon request.

Reviews, decision letters, and point-by-point responses associated with peer-review at Life Science Alliance will be published online, alongside the manuscript. If you do want to opt out of having the reviewer reports and your point-by-point responses displayed, please let us know immediately.

IMPORTANT: If you will be unreachable at any time, please provide us with the email address of an alternate author. Failure to respond to routine queries may lead to unavoidable delays in publication.

Scheduling details will be available from our production department. You will receive proofs shortly before the publication date. Only essential corrections can be made at the proof stage so if there are any minor final changes you wish to make to the manuscript, please let the journal office know now.

DISTRIBUTION OF MATERIALS:
Authors are required to distribute freely any materials used in experiments published in Life Science Alliance. Authors are encouraged to deposit materials used in their studies to the appropriate repositories for distribution to researchers.

You can contact the journal office with any questions, contact@life-science-alliance.org

Again, congratulations on a very nice paper. I hope you found the review process to be constructive and are pleased with how the manuscript was handled editorially. We look forward to future exciting submissions from your lab.

Sincerely,

Eric Sawey, PhD
Executive Editor
Life Science Alliance
http://www.lsajournal.org
